# Investigating the genetic components of tuber bruising in a breeding population of tetraploid potatoes

**DOI:** 10.1186/s12870-023-04255-2

**Published:** 2023-05-05

**Authors:** Olivia Angelin-Bonnet, Susan Thomson, Matthieu Vignes, Patrick J. Biggs, Katrina Monaghan, Rebecca Bloomer, Kathryn Wright, Samantha Baldwin

**Affiliations:** 1 The New Zealand Institute for Plant and Food Research Limited, Palmerston North, 4442 New Zealand; 2 The New Zealand Institute for Plant and Food Research Limited, Christchurch, 8140 New Zealand; 3 School of Mathematical and Computational Sciences, Massey University, Palmerston North, 4412 New Zealand; 4 School of Natural Sciences, Massey University, Palmerston North, 4412 New Zealand; 5 School of Veterinary Science, Massey University, Palmerston North, 4412 New Zealand

**Keywords:** *Solanum tuberosum*, Tuber bruising, GWAS, Differential expression, Systems biology

## Abstract

**Background:**

Tuber bruising in tetraploid potatoes (*Solanum tuberosum*) is a trait of economic importance, as it affects tubers’ fitness for sale. Understanding the genetic components affecting tuber bruising is a key step in developing potato lines with increased resistance to bruising. As the tetraploid setting renders genetic analyses more complex, there is still much to learn about this complex phenotype. Here, we used capture sequencing data on a panel of half-sibling populations from a breeding programme to perform a genome-wide association analysis (GWAS) for tuber bruising. In addition, we collected transcriptomic data to enrich the GWAS results. However, there is currently no satisfactory method to represent both GWAS and transcriptomics analysis results in a single visualisation and to compare them with existing knowledge about the biological system under study.

**Results:**

When investigating population structure, we found that the STRUCTURE algorithm yielded greater insights than discriminant analysis of principal components (DAPC). Importantly, we found that markers with the highest (though non-significant) association scores were consistent with previous findings on tuber bruising. In addition, new genomic regions were found to be associated with tuber bruising. The GWAS results were backed by the transcriptomics differential expression analysis. The differential expression notably highlighted for the first time the role of two genes involved in cellular strength and mechanical force sensing in tuber resistance to bruising. We proposed a new visualisation, the HIDECAN plot, to integrate the results from the genomics and transcriptomics analyses, along with previous knowledge about genomic regions and candidate genes associated with the trait.

**Conclusion:**

This study offers a unique genome-wide exploration of the genetic components of tuber bruising. The role of genetic components affecting cellular strength and resistance to physical force, as well as mechanosensing mechanisms, was highlighted for the first time in the context of tuber bruising. We showcase the usefulness of genomic data from breeding programmes in identifying genomic regions whose association with the trait of interest merit further investigation. We demonstrate how confidence in these discoveries and their biological relevance can be increased by integrating results from transcriptomics analyses. The newly proposed visualisation provides a clear framework to summarise of both genomics and transcriptomics analyses, and places them in the context of previous knowledge on the trait of interest.

**Supplementary Information:**

The online version contains supplementary material available at 10.1186/s12870-023-04255-2.

## Background

Genome-wide association studies (GWAS) use statistical models to detect causal regions in the genome that control a trait of interest. While such genomic association studies have been extensively applied to diploid species, more work is still required for polyploid species, owing to their higher genetic complexity. Among them, the potato *Solanum tuberosum* is a tetraploid crop of great economic importance, accounting for 4% of the global crop production, with 0.37 billion tonnes produced around the globe in 2021 [1]. As a consequence, a better understanding of its agronomic and quality traits is crucial for breeding programmes, in order to develop improved lines. This has been hindered by the complex genetic architecture of these traits, the high heterozygosity of the crop, and the lack of genomic and genetic tools and technologies dedicated to polyploid genetic studies. Previous studies on potato genetics have made use of diploid species of potatoes [2–5], biparental populations [2–4, 6], restricted the analysis to dominant markers or diploidised the markers’ dosage (i.e., the number of alternate alleles carried by a sample) in order to use the diploid tools available [7], or focused on candidate genes for the association analysis [8–14]. Despite these drawbacks, numerous studies have investigated the genetic basis of important potato traits, notably through the use of GWAS [11, 15–18]. Recently, an R package for GWAS analysis of tetraploid organisms, GWASpoly, was proposed [16]. GWASpoly implements several genetic models to explain the impact of a marker’s dosage on the phenotype. Importantly, it allows the inclusion of population structure in the model in order to correct for the impact of possible subpopulations on the resulting marker scores. Unaccounted for population structure can lead to spurious marker-trait associations, due to differences in trait and allele frequencies distribution across the subpopulations [19, 20]. This was notably investigated in association studies performed on the potato [16, 18]. In addition, while association studies are most informative when performed on a diverse population with a large variability for the trait under study, genomic data collected within breeding programmes provide an interesting source of information that could be harnessed in the context of GWAS studies, provided that the traits investigated are not directly subject to selection. Moreover, the markers identified in this way would be directly relevant to the breeding programme and could thus potentially be used subsequently for marker-assisted selection. Therefore, it is of interest to assess whether breeding populations can be used to discover genotype-phenotype relationships through GWAS.

In a complementary approach to genomic analyses, RNA sequencing technologies enable the investigation of the transcriptome, i.e. of the expression of all genes in an organism. Transcriptomics studies can identify key genes involved in biological mechanisms of interest, typically through differential expression analysis. RNA sequencing has been used for the study of both the diploid and more recently the tetraploid potato, mostly in the context of crop improvement. For example, transcriptomics analyses identified genes affecting tuber flesh colour [21], drought resistance [22, 23], cold response during post-harvest storage [24], infection by pathogen *P. infestans* [25], salt tolerance [26], or traits of economic interest such as yield or growth rate [27] in tetraploid cultivars or breeding lines. For a list of recent transcriptomics studies on the potato, we refer the reader to [28, 29]; and a description of the different steps of RNA sequencing analysis specifically for potato data, along with example datasets and recommendations can be found in [30]. Recently, RNA sequencing has also been used to conduct marker discovery in a population of 181 tetraploid cultivars for genomic selection [31]. In an effort to improve future transcriptomics analyses, Petek and collaborators constructed a potato pan-transcriptome, which identify genes that are shared across or unique to four tetraploid cultivars, and which improves upon current potato genomic resources for transcriptome annotation [32].

In this study, we focused on unravelling the genetic components of tuber bruising. Tuber bruising, also termed enzymatic or blackspot bruising, is the browning of the tuber flesh below the skin following a mechanical shock [9, 33]. Cell disruption through mechanical damage leads to the oxidation of phenolic compounds by polyphenol oxidases (PPO), leading to the formation of melanin pigments that give a brown colouration to the flesh [34]. Cellular damage through mechanical impact is common throughout all stages of potato handling, from harvesting to shipping. Tuber bruising is an important quality trait as it affects the flesh appearance, quality and flavour, and thus affects the tubers’ fitness for sale [33]. The annual cost of tuber bruising to the potato industry is estimated at several millions of US dollars in the US and tens of millions of pounds in the UK [35]. The development of potato lines that are more resistant to bruising is therefore a desirable objective for breeding programmes, rendering the genetic analysis of this trait an important task. Previous studies have identified several candidate causal genes and Quantitative Trait Loci (QTL) regions for the bruising phenotype. One such study focused on genes from the PPO family, in particular on the *POT32* gene, which is a major PPO gene expressed in potato tubers, and other genes involved in cell structure and shape, membrane stability, as well as carbohydrate metabolism [9]. Some of these candidate genes were identified by first comparing the concentration of the corresponding proteins between tubers with different bruising susceptibility [36]. Urbany et al. [9] developed markers from the sequences of these candidate genes. Several of these markers were found associated with tuber bruising, notably those constructed from *LipIII27* (located on chromosome 2), *4CL* and *PHO1A* (chromosome 3), *HQT* (chromosome 7) and PPO genes *POT32* and *potpoloxA* (chromosome 8). The influence of PPO enzymes on tuber bruising was already established by Werij et al. [2], who uncovered a QTL region on chromosome 8 that co-localised with the *POT32* gene sequence. Additional candidate genes were later identified on chromosomes 5, 8 and 12 [5].

We started by making use of a high-density capture-based (capture-seq) genotyping platform to carry out a GWAS analysis on a panel of half-sibling populations (hereafter referred to as a half-sib panel) to uncover regions of interest for the bruising phenotype. This half-sib panel consisted of individuals obtained from an early stage of the selection pipeline in a breeding programme. We augmented the GWAS results with RNA sequencing data, in order to investigate gene expression in response to tuber bruising, and to complement the GWAS results. The contributions of the article are as follows: (i) we demonstrate the use of GWAS on a breeding population of related autotetraploid individuals with a complex population structure that is subject to selection, (ii) we investigate the impact of the genetic model used in the association analysis, (iii) we show how such GWAS analysis can be enriched with complementary transcriptomics data, and (iv) we propose a new visualisation, the HIDECAN plot (acronym for high-scoring markers, differentially expressed genes, and candidate genes), to summarise and integrate GWAS results with transcriptomics differential expression analysis and previous knowledge about the genetic components involved. This study bridges the gap between genotype and phenotype by combining genomics, transcriptomics and phenotype data to ultimately unravel the mechanisms of potato tuber bruising.

## Results

### Genotype by sequencing using capture sequencing

Genotype by sequencing via capture sequencing was performed on tetraploid potatoes from a half-sib panel. The panel was obtained by crossing two parents of interest, Crop20 and its progeny Crop52, selected for their storage and processing qualities, with a number of other varieties (Table 1). The varieties used as second parents consist of both related and unrelated individuals (see Fig. S1-S4 for pedigree of genotyped parents as recovered from [37]). Note that the selection of the parents was not based on bruising resistance qualities of the varieties. For the genotype by sequencing, baits were designed to capture exonic regions across the genome. Initial variant calling yielded 1,388,205 variants. Amongst them, 1,205,998 heterozygous biallelic single nucleotide polymorphisms (SNPs – 86.9% of all variants) were retained for further analysis. SNPs were filtered out based on missingness of > 10%, quality and minor allele frequency of < 0.01. This yielded a total of 454,247 variants (37.7% of the biallelic SNPs) that passed the filtering step. This number is not unexpected given the high heterozygosity of the tetraploid potato and the population design, in which parents with varied genotypes were crossed. In addition, out of the 178 genotyped samples, two of them did not meet the criteria of less than 10% of missing data and were consequently discarded, leaving 176 samples with genotype information (158 progeny genotypes and 13 parent genotypes, with some parents measured through several biological or technical replicates).

**Table 1:** Cross design of the half-sib panel. For each cross between two parents, the number of progeny genotypes used for the GWAS analysis are presented, as well as the number of progeny genotypes used in the low- and high-bruising groups for the transcriptomics differential analysis. Some progeny genotypes with transcriptomics and phenotype data were not used in the differential analysis as their bruising score did not meet the criteria of the low- or high-bruising group. Only a subset of Crop52 progeny was used for the transcriptomics analysis

			Number of progeny used for GWAS analysis	Number of progeny with transcriptomics and phenotype data			
Cross ID	Parent 1	Parent 2	Total	Total	Low bruising group	High bruising group	Not used for DE analysis
2114	Crop20	V390	7	0	0	0	0
2132	Crop20	‘964/8’	5	0	0	0	0
2134	Crop20	‘Summit Russet’	9	0	0	0	0
2135	Crop20	VR808	3	0	0	0	0
2136	Crop20	Crop37	5	0	0	0	0
2138	Crop20	LT1	5	0	0	0	0
2140	Crop20	810_7	6	0	0	0	0
2121	Crop52	V390	11	11	1	6	4
2155	Crop52	‘Admiral’	10	10	5	2	3
2158	Crop52	‘Dolce Vita’	13	13	6	2	5
2159	Crop52	‘Driver’	16	16	7	5	4
2164	Crop52	‘Karaka’	4	0	0	0	0
2165	Crop52	‘Crop18’	10	9	2	7	0
2166	Crop52	‘Markies’	3	0	0	0	0
2167	Crop52	‘Mondial’	2	0	0	0	0
2168	Crop52	‘Moonlight’	7	7	2	4	1
2169	Crop52	‘Crop33’	5	0	0	0	0
2170	Crop52	‘Red Rascal’	8	8	7	0	1
2172	Crop52	‘Summit Russet’	7	7	2	2	3
2173	Crop52	VR808	2	0	0	0	0
2175	Crop52	Crop9	8	8	3	3	2
2177	Crop52	Crop37	6	6	2	2	2
2183	Crop52	‘Crop58’	6	5	4	0	1
**Total**			158	100	41	33	26

As expected, variant density along the chromosomes correlates with bait density, and decreases near the centromeres (Fig. S5). This is because baits were designed to capture exons of annotated genes, therefore they preferentially target regions with high exon density, and are thus less abundant near the centromeres where there are less protein-coding genes.

Strong linkage disequilibirum (LD) due to physical proximity can be expected between variants detected with the same bait, as well as between variants obtained from baits that are located close to each other along a chromosome. Therefore, in order to reduce the impact of LD on subsequent analyses and to reduce the number of variants to analyse, LD pruning was performed. Variants were recursively removed using a sliding window of 100kb, and a relaxed threshold of 0.5 on the $r^2$ values between pairs of variants was used. This led to a final dataset containing 72,847 variants (16% of all filtered variants – Fig. S6).

### Population structure amongst the genotyped potatoes

**Fig. 1:**
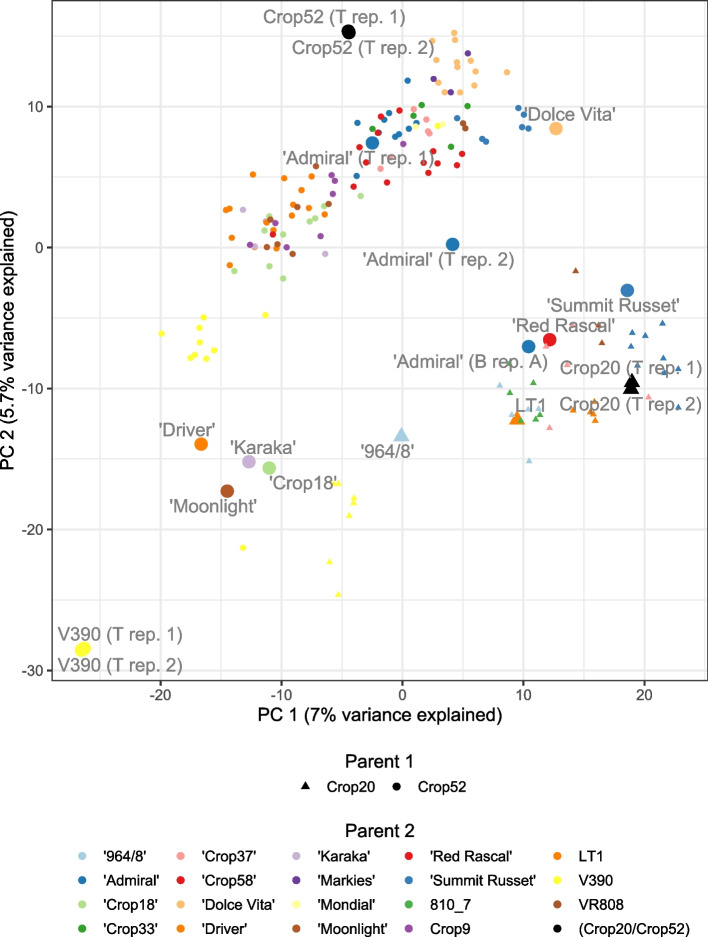
PCA plot of the first and second components of a PCA applied to variants’ most probable dosage for 176 samples. The name of the parent samples (large points) is indicated next to the corresponding point, while progeny samples are indicated with smaller points. For parent samples, the suffix ‘T Rep. *i*’ represents technical replicates, while ‘B Rep. *A*’ represents biological replicates. For the progeny samples, the shape of the points represent the first parent (i.e. Crop20 or Crop52), and the colour the second parent

The results of a Principal Component Analysis (PCA) performed on variant dosage information for the 176 samples are shown in Fig. 1 (see also Fig. S7-S9). The first and second principal components separate the Crop20 and Crop52 progeny, positioned in the lower right and upper left triangles of the plot, respectively, while also segregating V390, ‘Driver’, ‘Karaka’, ‘Crop18’ and ‘Moonlight’, from the other parents. The progeny samples from a cross between two genotyped parents are positioned half-way between the two parents in the PCA plot, which is expected as they share half of the genetic material of each parent. The technical replicates available for parent samples overlap, which indicates good quality of the data, except for the ‘Admiral’ samples, probably due to sample misslabelling.

**Fig. 2:**
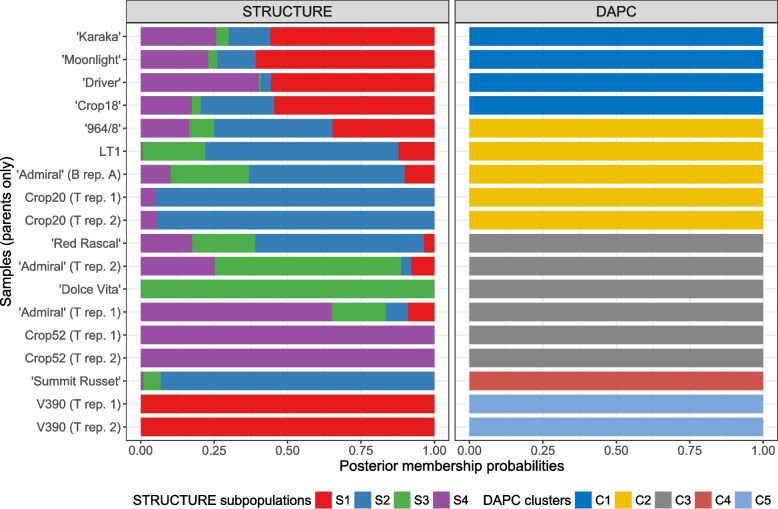
Population structure uncovered with STRUCTURE and DAPC for parent samples. The posterior membership probabilities of the samples are displayed for the four subpopulations identified by STRUCTURE (left panel) and for the five clusters identified by DAPC (right panel). B rep.: biological replicate; T rep.: technical replicate

The software STRUCTURE was used to uncover the population structure among the 176 samples. Four subpopulations were identified as the optimal setting (see Fig. S10), and five runs of STRUCTURE with the number of subpopulations set to four led to very similar results. The main output of STRUCTURE is the posterior membership probability profile of each sample, i.e., the probability that the sample belongs to each of the four detected subpopulations. This informs about the possible admixture of the samples, in the case where the membership probability of a sample is not zero for more than one subpopulation. Overall, the subpopulations identified are consistent with the known pedigree of the samples and with the PCA results (Fig. 2). V390, ‘Dolce Vita’ and Crop52 are assigned exclusively (posterior membership probability > 0.99) to subpopulations S1, S3 and S4, respectively. Crop20 is assigned mainly to subpopulation S2, with a non-null membership probability (0.05) for subpopulation S4, consistent with its parental relationship with Crop52. The remaining parents are detected as an admixture of these subpopulations. In particular, varieties ‘Moonlight’, ‘Karaka’, ‘Driver’ and ‘Crop18’, which share V394 as a common parent, are assigned a similar posterior membership probability distribution. This is expected given their relatedness. Interestingly, for these four parents, the highest membership probability corresponds to the V390 subpopulation. Even though V390 and V394 originate from the same breeding programme, it is not known whether they share a common lineage; these results tend to indicate that it is the case. ‘Red Rascal’ yields a large posterior membership probability for the Crop20 subpopulation, consistent with the fact that the two varieties are related (Fig. S1). Note that the ‘Admiral’ replicates each have markedly different membership probability profiles, which provides evidence of possible sample mislabelling. Unsurprisingly, the membership probability profile of each progeny sample is an equal mix of these of the corresponding parents (Fig. S11).

Discriminant Analysis of Principal Components (DAPC) was also used to investigate population structure. The analysis revealed five sample clusters as the optimal setting (Fig. S12-S13), where the clusters inform us about different subpopulations within the samples (Fig. 2). One of the outputs of DAPC is a measure similar to the posterior membership probability computed with STRUCTURE; however, it mainly reflects the attribution of the samples to the different clusters and is less informative about possible admixture. Again, the clusters obtained are mostly consistent with the known pedigree of the parent varieties. Cluster C1 gathers the V394 progeny, i.e. ‘Moonlight’, ‘Crop18’, ‘Karaka’ and ‘Driver’. ‘964/8’, ‘Red Rascal’, LT1, Crop20 and one of the ‘Admiral’ replicate are grouped in cluster C2. This is surprising because, while ‘Red Rascal’ and Crop20 are related, ‘964/8’ is instead more closely related to ‘Karaka’ (its parent), and thus would be expected to cluster with the latter. However, this is consistent with the PCA results, in which ‘964/8’ lies half-way between ‘Karaka’ and the LT1/Crop20/‘Red Rascal’ cluster on the first principal component. ‘Summit Russet’ and V390 are assigned to cluster C4 and C5, respectively. ‘Dolce Vita’ and Crop52 are grouped in cluster C3 with DAPC, together with two of the three ‘Admiral’ replicates. With DAPC, the progeny samples of each cross are all grouped with one of the parents (Fig. S14).

A comparison of the STRUCTURE and DAPC results for all samples is presented in Fig. S15. Overall, even though there is not necessarily a one-to-one correspondence between the STRUCTURE subpopulations and the DAPC clusters, the two methods are mostly in agreement. For example, STRUCTURE subpopulation S1 and DAPC cluster C5 both correspond to V930 and its progeny; DAPC cluster C1 groups all parents with a similar STRUCTURE subpopulation admixture pattern and their progeny. On the other hand, ‘Crop52’ and ‘Dolce Vita’ are assigned to two different subpopulations with STRUCTURE (S3 and S4, respectively), while they are both clustered in DAPC cluster C3.

### Genome-wide association study of tuber bruising

**Fig. 3:**
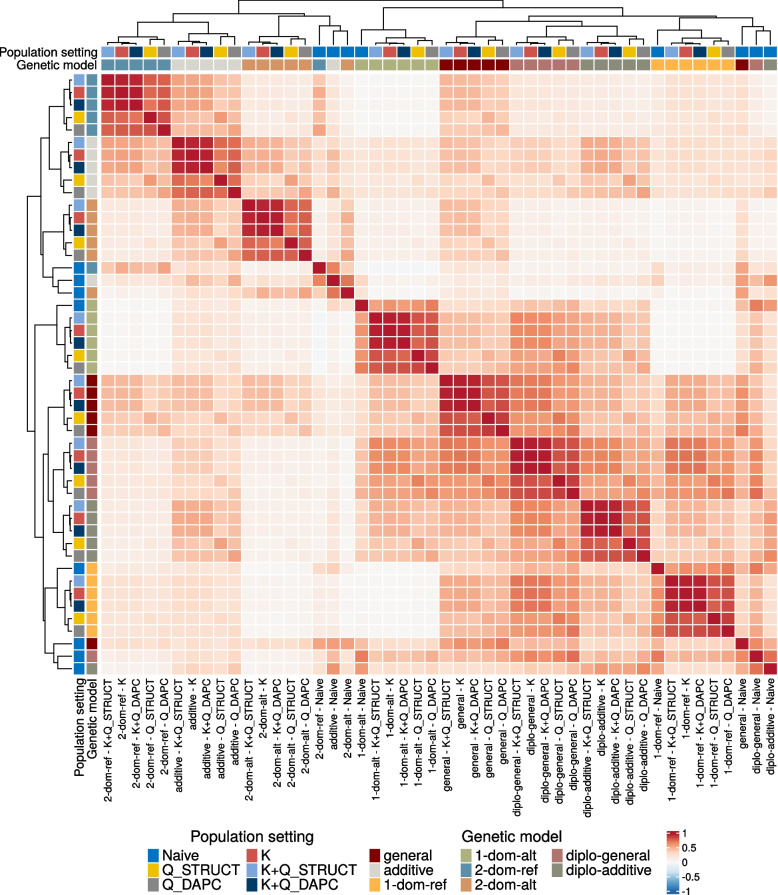
Heatmap of the correlation between GWAS marker scores obtained with different population settings and genetic models. For each pair of GWAS models, each with a different combination of population setting and genetic model, the correlation between the markers scores obtained with these two models is computed. In the heatmap, the rows and columns correspond to the different GWAS models, and darker shades of red indicate higher correlation values. The GWAS models (i.e. rows and columns) are clustered according to their correlation with other models. The coloured squares on the left of each row and at the top of each column showcase the population setting (outer squares) and the genetic model (inner squares) used in the corresponding GWAS model

Bruising was assessed for the 158 progeny samples via mechanical impact, followed with visual bruise scoring 24h after impact (Fig. S17 and Table S3). A GWAS analysis was performed on these progeny samples. Eight different genetic models (describing the effect of a marker’s dosage on the phenotype) and six different population settings (correcting for individual relatedness and/or population structure) were considered, yielding 48 sets of marker scores (see Methods section for details). Marker scores obtained with the same genetic model were found to be highly positively correlated, with the exception of scores obtained with the naive population setting (i.e. no correction for population structure), which were more similar between them than to scores obtained with a same genetic model but a different population setting (Fig. 3). In addition, for a same genetic model, marker scores obtained with the K only (i.e. including a kinship matrix in the model) or one of the K + Q models (which include both a kinship matrix as well as the population membership probabilities from either STRUCTURE or DAPC as covariates) were very highly correlated ($\ge 0.95$), and the marker scores obtained with the Q only models (population membership probabilities as covariates but no kinship matrix) were also highly correlated ($\ge 0.74$). In particular, the highest correlations were found between the K only and the K + Q$_\text {DAPC}$ models, indicating that adding the population structure uncovered with DAPC in the model brought very little information above what the kinship matrix revealed. On the contrary, marker scores were in general only modestly correlated between different genetic models. The highest correlations were obtained between the diplo-general model and the general, diplo-additive and simplex models. The two duplex models yielded scores that were moderately correlated with those from the additive and general model, while they had little to no correlation with the scores from other genetic models.

**Fig. 4:**
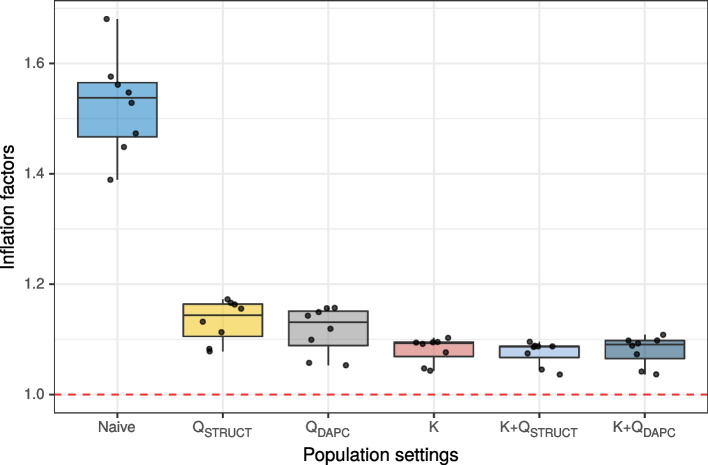
Inflation factors of marker scores for different GWAS population settings. Each point represents a different GWAS model with a specific population setting and genetic model

The ability of each population setting to control for false positives due to population structure in the resulting marker scores was evaluated by computing an inflation factor [16] for each GWAS model (Fig. 4). This inflation factor describes the deviation of the observed marker scores from their expected value under the null hypothesis of no association with the phenotype. Under the assumption that most markers are not associated with tuber bruising, it is expected that the inflation factor should be close to one. As expected, the naive population setting yielded elevated inflation factors (with a mean inflation factor of 1.53). From the Q models, using the results from DAPC was slightly more efficient at reducing inflation factors than using the STRUCTURE results (mean of 1.12 for Q$_\text {DAPC}$ model vs 1.13 for Q$_\text {STRUCT}$ model). The K only and K+Q models produced very similar inflation factors (mean of 1.08 for K and K+Q models). For each population setting, the average inflation factor across all genetic models was computed, and the population setting yielding the average inflation factor closest to one was retained as the best setting to correct for population structure. In this case, the K + Q$_\text {STRUCT}$ was retained, although the reduction in inflation score compared to the K only model was minimal. The QQ plots of the marker scores for the different genetic models obtained with the K + Q$_\text {STRUCT}$ model are presented in Fig. S18.

A significance threshold for marker’s *p*-values was computed by correcting for multiple testing using a false discovery rate (FDR) scheme. After correction for multiple testing, five markers were considered as significantly associated with the phenotype: at 12.8Mb on chromosome 1, 5Mb on chromosome 7, and at 45.9Mb, 46Mb and 49.1Mb on chromosome 8. The marker on chromosome 1 was assigned a significant score with four genetic models (general, diplo-general, diplo-additive and 1-dom-alt); the other four were only significant with one genetic model (2-dom-ref for marker at 45.9Mb on chromosome 8, 1-dom-ref for the others). Interestingly, the two significant markers on chromosome 8 around 46Mb were very close to genes encoding PPOs (PGSC0003DMG400018914, PGSC0003DMG400018917), including the *POT32* gene (PGSC0003DMG400018916); such genes code for the enzymes responsible for triggering the production of melanin pigments that gives the bruising colour. Furthermore, the position of 37 markers with a non-corrected *p*-value below $1\times 10^{-4}$ (i.e. GWAS score above 4 – Table S1) was also investigated, and compared to candidate genes and genomic regions found associated with tuber bruising from the literature (Table 2). One high-scoring marker located at 50.7Mb on chromosome 7 was found within 1Mb of a phosphate adenylyltransferase gene (PGSC0003DMG400031084), while two high-scoring markers on chromosome 8 between 3.2 and 3.4Mb were close to several patatin genes. In both cases, the proteins associated with these genes were found to be differentially expressed between tubers with low- and high-bruising susceptibility in [36]. In addition, one high-scoring marker on chromosome 3 around 52Mb was close to a proteinase inhibitor type-2 gene (PGSC0003DMG400004547), whose protein was also identified by Urbany et al. [36] as differentially abundant between high- and low-bruising tubers. Several other high-scoring GWAS markers were found within 5Mb of candidate genes previously identified. Other markers with high GWAS scores were not found near previously detected QTLs; notably around 6 - 8Mb on chromosome 1 and around 0.8 - 1 Mb on chromosome 11.

**Table 2:** List of candidate genes identified as potentially involved in tuber bruising in previous studies. The References column cites the article in which genes were found related to bruising, as well as the publication in which the gene was identified (if different)

Gene annotation	Abbreviation	Gene ID (PGSC0003DMG)	Chromosome	Position (MB)	References
$\alpha$-glucan phosphorylase L-type	PHO1A	400007782	ST4.03ch00	38.54	[5, 9]
Triacylglycerol lipase III	LIPIII27	400023182	ST4.03ch02	10.09	[5, 9]
Patatin 3	PAT3	400000766	ST4.03ch02	23.7	[36]
Phospholipase A1	PLA1	400010221	ST4.03ch02	32.41	[36]
Phospholipase A1	PLA1	401031759	ST4.03ch02	33.04	[36]
Hydroxyproline-rich glycoprotein family protein	Stl024	400010074	ST4.03ch02	44.53	[9, 17]
Fibrillarin homologue	Stl013	400010119	ST4.03ch03	43.66	[9, 17]
Aspartic protease inhibitor 8	CDI	400009511	ST4.03ch03	44.04	[36]
4-coumarate CoA ligase	4CL	400003155	ST4.03ch03	47.07	[9, 17]
Kunitz-type enzyme inhibitor *S9C11*	KTEI	400010147	ST4.03ch03	49.39	[36]
Cysteine protease inhibitor 1	STKTI	400010143	ST4.03ch03	49.55	[36]
Cysteine protease inhibitor 1	CPI1	400010139	ST4.03ch03	49.64	[36]
Kunitz-type proteinase inhibitor	KTPI	400009512	ST4.03ch03	49.93	[36]
Aspartic protease inhibitor 5	PKPI-A5	400009513	ST4.03ch03	49.93	[36]
Proteinase inhibitor type-2 P303.51	PIN2K	400004547	ST4.03ch03	50.06	[36]
4-coumarate CoA ligase 2	4CL2	400014223	ST4.03ch03	57.57	[9]$^*$
Ci21A protein	CI21A	400006662	ST4.03ch04	60.2	[36]
Annexin p34	AN34	400017714	ST4.03ch04	63.04	[36]
Phenylalanine ammonia-lyase	PAL	400005492	ST4.03ch05	36.35	[5]
Peroxidase	PEROX	400005279	ST4.03ch05	42.52	[5]
Hydroxycinnamoyl quinate CoA transferase	HQT	400011189	ST4.03ch07	1	[5, 9]
Glucose-1-phosphate adenylyltransferase small subunit, chloroplastic/amyloplastic	AGPB1	400031084	ST4.03ch07	51.45	[36]
Patatin 05	PAT1-K1	400008749	ST4.03ch08	1.44	[36]
Patatin-2-Kuras 4	PAT2-K4	400014104	ST4.03ch08	1.47	[36]
Patatin-04/09	PAT0409	402017090	ST4.03ch08	1.56	[36]
Patatin group O	PATO	400029247	ST4.03ch08	1.65	[36]
Lipoxygenase	LOX1-ST-2	400020999	ST4.03ch08	6.05	[36]
Catechol oxidase B, chloroplastic	COB	400029575	ST4.03ch08	45.63	[36]
Polyphenol oxidase	PPO	400029576	ST4.03ch08	45.68	[9]$^*$
Polyphenol oxidase	PPO	400018924	ST4.03ch08	45.78	[9]$^*$
Polyphenol oxidase	PPO	400018919	ST4.03ch08	45.79	[9]$^*$
Polyphenol oxidase B, chloroplastic	PPO	400018925	ST4.03ch08	45.84	[9]$^*$
Polyphenol oxidase isoform *POT32*	POT32	400018916	ST4.03ch08	45.87	[9, 36]
Polyphenol oxidase A, chloroplastic	PPO	400018917	ST4.03ch08	45.87	[9]$^*$
Polyphenol oxidase	PPO	400018914	ST4.03ch08	45.89	[9]$^*$
Polyphenol oxidase	PPO	400018913	ST4.03ch08	45.91	[9]$^*$
Glucose-1-phosphate adenylyltransferase	AGPB	400046891	ST4.03ch12	1.23	[36]
Triacylglycerol lipase III	LIPIII27	400007805	ST4.03ch12	1.49	[5, 9]
Triacylglycerol lipase III	LIPIII	400028858	ST4.03ch12	53.25	[5]
Miraculin	KTI-D	400008546	ST4.03ch12	55.09	[36]

* Gene IDs obtained from EnsemblPlant

### Transcriptomics differential expression

In order to assess how change in gene expression in response to bruising differs between tubers with different bruising susceptibilities, transcriptomics data were generated for a subset of progeny samples with Crop52 as parent (see Table 1), two hours after bruising, at the site of the bruising. The expression of 25,163 genes was obtained by RNA sequencing (after quality filtering of genes). A differential expression analysis was performed between samples with low bruising and samples with high bruising. Out of the 100 samples with both gene expression data and mean bruising score, 41 samples with a mean bruising score of one or less (low bruising group) were compared with 33 samples with a bruising score of two or more (high bruising group). Fifty-seven genes were found significantly differentially expressed (DE) after FDR correction (with a significance threshold of 0.05), including 30 genes found to be upregulated in high-bruising tubers, and 27 genes downregulated (Table S2). Ten of the DE genes were annotated as “gene of unknown function”. The Gene Ontology terms found significantly enriched for DE genes via a functional class scoring analysis are presented in Table 3. The enrichment results highlight an over-representation of protein modifications and protein kinase activity amongst the differentially expressed genes. Indeed, six differentially expressed genes code for diverse kinases, and three other genes are involved in protein ubiquitination. These three genes are a ring finger protein-coding gene (PGSC0003DMG400012137), a gene coding for an ubiquitin carboxyl-terminal hydrolase isozyme L3 (PGSC0003DMG400026229) and a gene from the phototropic-responsive NPH3 family (PGSC0003DMG400017655). The differential expression of kinases and other genes involved in protein modification is consistent with an activation of stress response upon bruising. Indeed, signalling pathways, notably kinase-dependent pathways, are activated in response to stress and propagate signals throughout the cell and to the nucleus, in order to trigger appropriate cellular responses [38]. For example, an increased expression of mitogen-activated kinases in response to wounding has been observed in potatoes [39].

**Table 3:** Gene Ontology (GO) terms enriched for differentially expressed genes. The number of genes associated with the each GO term is presented in the column ‘Number of genes’

GO domain	GO ID	GO term	Adjusted *p*-value	Number of genes
Molecular function	GO:0005524	ATP binding	0.006	2,010
Molecular function	GO:0005515	Protein binding	0.006	2,578
Biological process	GO:0006468	Protein phosphorylation	0.014	1,110
Molecular function	GO:0004672	Protein kinase activity	0.014	1,088
Molecular function	GO:0016740	Transferase activity	0.043	1,963
Molecular function	GO:0004674	Protein serine/threonine kinase activity	0.043	654

In addition, several other differentially expressed genes can be linked to mechanisms of plant stress response. For example, an ethylene-responsive AP2/ERF domain-containing transcription factor (PGSC0003DMG400020625) was found to be upregulated in potatoes with high bruising scores; the AP2/ERF transcription factors family is a key regulator of plant stress response [40, 41]. A phosphate-induced BZIP-domain containing transcription factor (PGSC0003DMG400025889) whose expression was also found to be increased in high-bruising tubers, shares a 78% nucleotide sequence identity with a phosphate-induced transcription factor in *Nicotania tabacum* (tobacco) involved in stress response through the abscisic acid (ABA) signalling pathway [42]. Similarly, the upregulated *UPA16* gene (PGSC0003DMG400033693), encoding a transmembrane sugar transporter protein, shares an 86% nucleotide sequence identity with a closely located gene encoding a protein with similar function that was found to be differentially expressed between potatoes with differing drought tolerance [43]. A gene encoding a dihydrodipicolinate synthase (PGSC0003DMG400019758), involved in plant stress response through the lysine biosynthesis pathway [44], as well as a heat-shock protein 70 (HsP70)-interacting protein-coding gene (PGSC0003DMG400012485) were both found to be downregulated in tubers presenting high bruising. The *StOPT8* gene (PGSC0003DMG400013297), encoding an oligopeptide transporter from the OPT family, involved in heavy metal stress response [45], was also downregulated in high-bruising tubers.

Several other genes involved in transmembrane transport mechanisms were amongst the differentially expressed genes, including two genes encoding proteins with oligopeptide or nitrate transmembrane transport activities (PGSC0003DMG400003820 and PGSC0003DMG400004795, respectively) that were found to be downregulated in high-bruising tubers. Also, a gene encoding a mechanosensitive ion channel domain-containing protein (PGSC0003DMG400009888) was downregulated in high-bruising tubers. Mechanosensitive ion channels are membrane proteins that translate mechanical forces applied to cells into cellular signals [46], and have not been found related to tuber bruising in previous studies. This provides a clue that some type of mechanosensing mechanism is involved in the tuber’s response and resistance to mechanical bruising. It has also been found in plants that some receptor-like kinases can detect cell wall damage [47, 48]. It is thus possible that some of the kinases found differentially expressed in the present study are related to such mechanisms, and that an increased ability of cells to detect (and thus react to) cellular damage caused by impact is linked to a difference in the tuber’s susceptibility to forming bruises.

Several genes involved in pathogen or disease resistance were also found to be differentially expressed, e.g. the Avr9/Cf-9 rapidly elicited protein 140-coding gene (PGSC0003DMG400010173 – expression reduced in high-bruising tubers), involved in the response to *Cladosporium fulvum*, which causes leaf mould in tomato [49], the disease resistance protein RGA3-coding gene (PGSC0003DMG400019806 – downregulated in high-bruising tubers) or the CC-NBS-LRR resistance protein-coding gene (PGSC0003DMG400008596 – upregulated at high bruising) [50]. This can be explained by the fact that stress-activated signalling pathways such as jasmonate or ABA pathways also trigger the expression of these disease resistance genes, in order to prepare the tuber for potential infections through wounded tissues [51]. An alternative explanation for the differential expression of these genes would be that the level of constitutive expression of some of these genes is higher in certain half-sibling families, in a process independent of bruising. Measurements on the same unwounded tubers would be necessary to assess the validity of this hypothesis.

Furthermore, we noted that two protease inhibitors were found to be differentially expressed. They include a peptidase inhibitor gene (PGSC0003DMG400010136) and an aspartic protease inhibitor gene (PGSC0003DMG400010129), both found on chromosome 3 between 43 and 50Mb, and encoding inhibitors of the Kunitz family. These results are in accordance with Urbany et al. [36], who detected several protease inhibitor proteins as differentially abundant between bruising-susceptible and bruising-resistant potato cultivars. Specifically, the genes encoding these proteins were found located within the *StKI* locus on chromosome 3 [52], which coincides with the protease inhibitor genes found differentially expressed in the present study. Urbany et al. [36] hypothesised that protease inhibitors could play a role in tuber bruising indirectly via the inhibition of enzymes involved in bruising-related pathways. Alternatively, these genes could be involved in stress response due to their role in signalling cascades or in defence against pathogens [53].

A gene encoding an anthocyanin synthase enzyme (PGSC0003DMG400022746) was found to be downregulated in tubers with high bruising scores. This is consistent with previous findings in which anthocyanin production was observed to decrease at the site of wounding in potatoes [54], leading to the discolouration of tuber skin.

Lastly, a gene coding for a caffeic acid O-methyl-transferase II protein, involved in the lignin biosynthesis pathway [55] but not yet linked to tuber bruising, was found to be downregulated in tubers with high bruising scores. Lignin is a cell wall polymer that forms a protective barrier in the periderm tissue layers of tubers [54]. It provides both physical strength to the tubers, as well as an antimicrobial layer that protects tubers from infection by exogenous pathogens. In the case of tuber bruising, physical resistance to mechanical impact is a key factor. Indeed, melanin pigments, which are responsible for the black colouration of the bruise at the impact site, are produced as a result of the oxidation of phenols by PPO enzymes. In normal conditions, PPOs and their substrates are kept apart in the cell through their localisation into different cellular compartments. However, upon mechanical impact, destruction of cellular compartments brings enzymes and substrates into contact, thus initiating the series of reactions ultimately leading to the production of melanin [34, 56]. Therefore, it has been observed that sensitivity to cellular breakage, which results in the release of PPOs and its substrates, could be an important factor of blackspot bruising susceptibility [34]. Consequently, the overexpression of caffeic acid O-methyltransferase II, likely resulting in increased production of lignin, could provide increased resistance of cell wall to mechanical wounding and thus reduced cellular destruction upon impact, ultimately resulting in a reduced production of melanin pigments, and therefore smaller and less pronounced bruises.

### Comparison of GWAS high-scoring markers and differentially expressed genes

Direct comparison of the GWAS high-scoring markers and DE genes highlighted one high-scoring marker located within a differentially expressed gene. The marker, located on chromosome 8 around 51.3Mb, coincides with an ubiquitin carboxyl-terminal hydrolase isozyme gene (PGSC0003DMG400026229) whose expression is found to be increased in high-bruising tubers. Two other differentially expressed genes, encoding a nitrate transporter (PGSC0003DMG400004795) and an anthocyanin synthase (PGSC0003DMG400022746), are located close to this high-scoring marker.

**Fig. 5:**
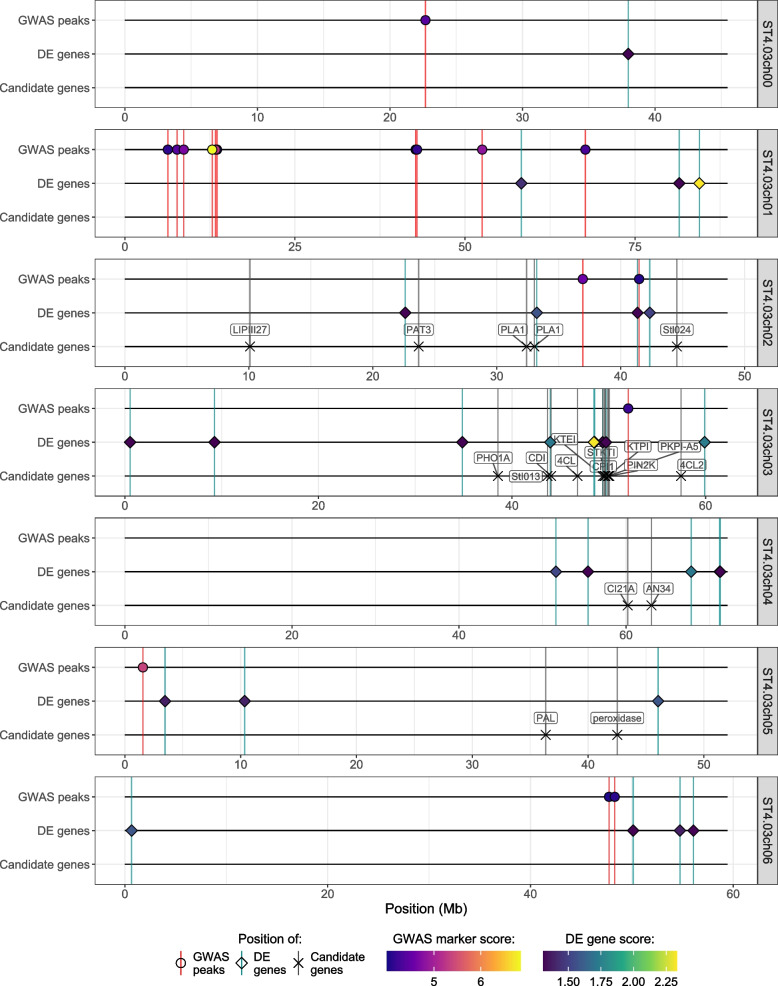
Genomic position of the GWAS high-scoring markers, differentially expressed genes and candidate genes extracted from the literature, along chromosomes 0 to 6 of the potato genome

**Fig. 6:**
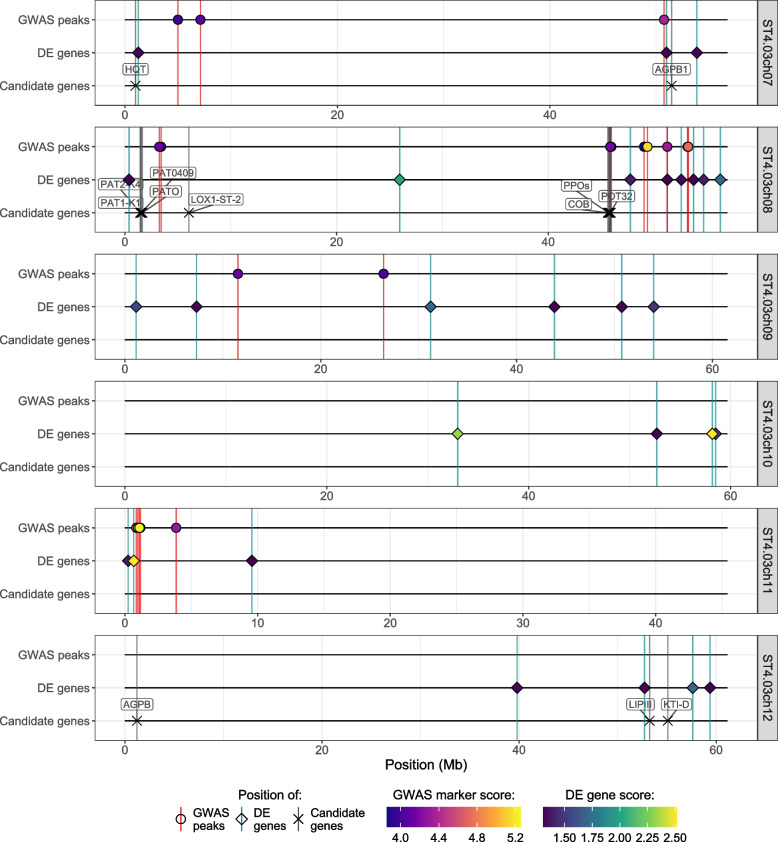
Genomic position of the GWAS high-scoring markers, differentially expressed genes and candidate genes extracted from the literature, along chromosomes 7 to 13 of the potato genome

In order to assess the overlap between the GWAS and transcriptomics analyses, we developed a new visualisation, called a HIDECAN plot (for high-scoring markers, differentially expressed genes, and candidate genes), shown in Figs. 5 and 6. The HIDECAN plot displays, along each chromosome of the studied organism, the genomic position of both high-scoring markers as well as differentially expressed genes. As can be seen in Figs. 5 and 6, several genomics regions contain both GWAS high-scoring markers and differentially expressed genes. On chromosome 2 between 41 and 42Mb, a high-scoring marker is found near two differentially expressed genes, including a transcription factor (PGSC0003DMG400021423) and a conserved gene of unknown function (PGSC0003DMG400026406). Similarly, a number of high-scoring markers and differentially expressed genes are found on chromosome 8 between 51 and 56Mb. The differentially expressed genes include two gene encoding proteins involved in ubiquitination processes (PGSC0003DMG400026229 and PGSC0003DMG400012137), a nitrate transporter gene (PGSC0003DMG400004795), a gene coding for an anthocyanin synthase (PGSC0003DMG400022746), and a potential transcription factor (PGSC0003DMG400012215). Lastly, between 0.2 and 1.2Mb on chromosome 11, two differentially expressed genes are closely located to a number of high-scoring markers. One of these differentially expressed genes encodes a caffeic acid O-methyltransferase II (PGSC0003DMG400013342), which, as noted above, is involved in the lignin biosynthesis pathway. The other codes for an oligopeptide transporter of the OPT family (PGSC0003DMG400013297).

## Discussion

### Accounting for population structure in GWAS analysis

When performing an association study on commercial crops such as potato, it is important to account for the fact that the cultivars used in the association panel can be related. This is of particular importance when using data from a breeding programme, in which the samples are highly related. To this end, we evaluated the ability of two methods, STRUCTURE and DAPC, to uncover relationships between the genotyped samples. In particular, we compared the results of the two methods with the known pedigree of the samples. We found the results of DAPC and STRUCTURE to be mostly, but not completely, in agreement with each other and with known pedigree of the samples.

Moreover, STRUCTURE was more informative than DAPC, due to its ability to model sample admixture. For example, posterior membership probabilities of the progeny samples obtained with STRUCTURE reflected the membership probability distribution of both parent samples. On the other hand, with DAPC, progeny samples were clustered with one of the parents, thus losing information about the clustering of the second parent. In addition, the STRUCTURE posterior membership probabilities of parent samples reflected their complex relationships better than the clustering membership probabilities obtained with DAPC. We hypothesise that such conclusions would hold for other breeding populations with complex design; however, for more unrelated individuals, such as samples from a diversity panel, DAPC results would probably be sufficient to uncover population structure.

In the GWAS results, we found that using a kinship model only (K model) was more effective at reducing the false positive inflation of the marker scores due to population structure than adding subpopulation membership probabilities as covariates only (Q models). Furthermore, adding to a kinship model the results of either STRUCTURE or DAPC as covariates in the model only minimally affected the resulting marker scores. In an association study on a potato diversity panel, Rosyara et al. [16] found that, for Q models, using the DAPC results was more efficient at reducing false positive inflation than using the STRUCTURE results. They ultimately selected a K+Q$_\text {DAPC}$ model as the best for reducing false positive inflation; however, they did not investigate a K+Q$_\text {STRUCT}$ model. In our case, the K+Q$_\text {STRUCT}$ model yielded an average inflation factor marginally closer to 1 than the K+Q$_\text {DAPC}$ model. This difference in conclusions likely arises from the difference in the type of population analysed. In Rosyara et al. [16], the study was performed on a diversity panel comprising varied wild and cultivated potato species. In contrast, our half-sib panel comes from a breeding programme with a complex multifactorial design. Therefore, it is not surprising that DAPC, which relies on clustering, is more efficient at detecting the different unrelated groups amongst samples from the diversity panel. On the contrary, it seems to struggle more when all samples are closely related. STRUCTURE on the other hand can better detect admixture and is thus able to detect subtle and complex relationships between the samples. Therefore, when working with genetic data obtained from a breeding programme, we recommend the use of STRUCTURE above DAPC to investigate population structure even though the consequences of using one over the other on the GWAS results were minimal.

### Statistically significant markers

The present GWAS analysis detected five markers as significantly associated with tuber bruising, after appropriate correction for multiple testing. This small number of significant results is not surprising, for two reasons. Firstly, the choice of parents used in the breeding programme as well as the selection applied to the progeny samples as part of the breeding programme, even though not directly targeting bruising potential, introduced bias in the analysis by affecting the distribution of observed phenotypes, and potentially by culling variants causal for bruising susceptibility in linkage disequilibrium with or overlapping variants causal for traits targeted by selection. For example, a QTL region on chromosome 1 for tuber bruising was found to coincide with a QTL for starch content [5], which is often a critical trait in breeding programmes, and thus likely to be selected for. This selection bias, however, provides an opportunity to detect variants with smaller effects on tuber bruising that otherwise would be masked by markers with larger effects.

Secondly, the relatively small sample size reduces the statistical power to detect markers in linkage disequilibrium with causal variants affecting tuber bruising. In addition, the small number of progeny from some of the crosses included in the analysis likely results in some variants that originate solely from one parent not being represented at a sufficient level to permit a proper assessment of its association with the phenotype. This second point is mitigated in part by the relationships between parents used for the crosses, which ensures that a large fraction of the variants are shared by several parents.

Nevertheless, we found that several markers with the highest (even though non-significant) GWAS scores coincided with known genomic regions or candidate genes previously found to be associated with tuber bruising. This is an exciting result, as it demonstrates that it is possible to use genomics data from breeding programmes, as opposed to large and extensive association panels, to perform GWAS. It also illustrates the importance in such studies of interpreting marker scores as an importance ranking, and to consider markers close to but under the significance threshold as they might represent biologically relevant signal. Lastly, this also supports the possibility that other markers with high but non-significant GWAS scores that were not found near the previously mentioned genomic regions (notably near a telomere on chromosomes 1 and 11) could point to causal variants more modestly associated with tuber bruising. This generates new hypotheses to guide future experiments on tuber bruising.

### Differentially expressed genes between high- and low-bruising tubers

While a GWAS analysis informs about genetic variation associated with a trait of interest, it does not provide information about the mechanisms through which these genetic variants operate. In addition, GWAS typically highlights genomic regions rather than specific genes, which makes it harder to reconstruct the molecular mechanisms linking genotype to phenotype. Measurements of gene expression, such as transcriptomics data, provide valuable information to complement the GWAS results. They offer a genome-wide view of the gene expression landscape of the organism under study, and allow to detect changes in the expression of individual genes associated with changes in the trait of interest. Therefore, differential expression results can identify candidate genes that explain how the genetic variations uncovered affect the considered phenotype.

Accordingly, we performed a differential expression analysis on transcriptomics data to compare gene expression in low- and high-bruising tubers. The results were consistent with known mechanisms of tuber bruising. In addition, a number of differentially expressed genes were associated with plant stress response. This is expected as tuber wounding is an abiotic stress [56], and therefore differences in tuber susceptibility to form bruises is likely to affect the dynamics and timing of tuber stress response, or the level of stress perceived by the tuber cells upon mechanical bruising (and thus the cellular response to this stress). In addition, as the transcriptomics data were gathered two hours after bruising, it reflects the early response of tubers to bruising. Two differentially expressed genes of particular interest were a gene encoding an enzyme central to the lignin biosynthesis and a gene encoding a mechanosensitive ion channel domain-containing protein. Neither of these genes were previously found related to tuber bruising.

### New visualisation to integrate GWAS and differential expression results

There is currently no satisfactory way to effectively represent in a same graph the results of both GWAS and differential expression analysis. Yet, such visualisation is essential to assess the overlap between the results of these two analyses. The only alternative currently is to report the physical distance between GWAS markers and the closest differentially expressed gene, which does not situate the information in a genome-wide context. As a consequence, we developed a new visualisation, the HIDECAN plot, to integrate in a single graph both GWAS and differential expression results, as well as candidate genes uncovered in previous studies. This new visualisation is a powerful approach to display and summarise the results of these two analyses, and to highlight genomic regions for which association with a trait of interest is detected at both the genomic and transcriptomic level. It also allows us to compare the results from the current study to existing knowledge about the genetic components of the trait of interest. The HIDECAN plot is especially effective in the case where capture-bait sequencing data, rather than whole-genome sequencing, is used for the GWAS analysis. Indeed, with capture-bait sequencing, measured markers in LD with true causal variants are likely to be physically located further away from the causal variants than when looking at whole-genome sequencing. This is because with capture-bait sequencing, we can only measure genomic variation within the genomic region targeted by the baits, which only covers a fraction of the genome. Therefore, if the baits only target coding sequences, which was the case in the present study, it will be very unlikely that a causal variant located in a non-coding region is directly measured. In this case, markers with high scores in the GWAS analysis might be linked to a true causal variant further away in the genome, rather than being reflective of the gene in or near where it is found.

With this visualisation, three genomic regions in particular were found associated with the bruising phenotype both at the genomic and transcriptomic levels: between 41 and 42Mb on chromosome 2, between 51 and 56Mb on chromosome 8, and between 0.2 and 1.2Mb on chromosome 11. This latter region in particular is of interest, with one of the two differentially expressed genes, encoding a caffeic acid O-methyltransferase II protein, associated with cellular mechanical strength. This is an exciting finding, as previous investigations on tuber bruising focused on or highlighted the metabolic components of bruising, but did not emphasise the physical aspect of cellular resistance or response to mechanical shock. The fact that a high-scoring marker was located close to this gene could be explained by the presence of a genetic mutation in a regulatory sequence affecting the expression of the gene. Again, this preliminary finding warrants further investigation in the role of this protein, and of lignin biosynthesis, in the resistance of tubers to bruising. Lastly, one marker was found directly within a differentially expressed gene encoding for a protein involved in ubiquitination processes. This could be due to the marker being in linkage disequilibrium with a variant in a regulatory region affecting the expression of the gene. We note that transcriptomics measurements were obtained only for progeny genotypes deriving from a cross involving Crop52. Therefore, it is possible that the impact on gene expression of markers of interest present only through Crop20 progeny have not been observed, and that further investigation reveals an even greater overlap between GWAS and differential expression results.

## Conclusion

In this study, we investigated the genetic components underlying tuber bruising in tetraploid potatoes (*Solanum tuberosum*), using genomics and transcriptomics data obtained on a half-sib panel from a breeding programme. First, population structure amongst the investigated samples was assessed, and the results highlighted the differences between two popular methods, STRUCTURE and DAPC. Following this, recommendations were made regarding the most appropriate tool to use depending on the type of population investigated. Second, a genome-wide association study was performed. Despite the small number of significant scores (with only five significant marker identified), the GWAS revealed biologically relevant signals, as some of the genomic regions with the highest (but non-significant) GWAS scores were in agreement with previous studies on tuber bruising. In addition, new regions of interest were unveiled that merit further investigation to assess their potential involvement in mechanisms of tuber bruising. This study demonstrates the use of breeding programme populations, as well as genotype by sequencing via exon capture, for GWAS.

Differential expression between low- and high-bruising tubers allowed us to complement the results from the association study. Amongst the 57 genes found to be differentially expressed, 13 were related to plant stress response and signalling, three to protein modification, and three to known groups involved in tuber bruising such as protease inhibitors. The implication of the physical aspect to tuber bruising, in addition to metabolic mechanisms, is underlined by the discovery of two differentially expressed genes related to cell mechanical strength, and to the membrane detection of physical force applied to the cells. These results were reinforced by the presence of a high-scoring marker near one of these differentially expressed genes. While additional studies are required to validate these results, these findings provide an unprecedented genome-wide overview of the genetic components of tuber bruising, and generate new hypotheses to guide further studies on potato bruising.

Lastly, we proposed the HIDECAN plot to integrate the results of GWAS and transcriptomics differential expression in the context of previous findings from the literature. This new visualisation provides a clear summary of the study’s findings, and highlights genomic regions whose association with the phenotype can be detected at both the genomic and transcriptomic level. We demonstrated the usefulness of the HIDECAN plot in the context of tuber bruising. The use of this new visualisation can be extended across organisms and traits to summarise and display results of genetic and transcriptomics analyses. With the increase in studies investigating both genomic and transcriptomics levels, we encourage a widespread adoption of such integrated visualisation when presenting the results of modern ‘omics analyses.

## Methods

### Plant material

Seeds were obtained by crossing parental potato lines (crossing design presented in Table 1). Progeny were culled and selected over three seasons.

### Phenotyping

Plants were grown at Lincoln, New Zealand (43.6400$^\circ$ S, 172.4862$^\circ$ E) in 2018 under local commercial growing conditions. Three tubers from each of two biological replicates were harvested for 158 progeny genotypes, and stored at 4°C for two weeks. Each tuber was bruised on two opposite sides by letting a lead weight fall through a one-metre pipe on the tuber. The zone of impact was recorded by applying ink to the lead weight prior to the bruising treatment. This bruising experiment provides a standardised method to replicate as closely as possible damage caused by handling and storage in commercial conditions. After 24 hours, tubers were sliced at the site of bruising, and an image was taken of the bruising for the three tubers of the two biological replicates per genotype. A bruising score was attributed to each tuber upon visual inspection of the images based on the bruising area, from zero (no visible bruising) to five (extensive bruise), according to a predefined visual scale (Fig. S16).

### Genotyping

#### Data collection and preprocessing

Samples were taken from young leaf tissue for 178 samples, which either had bruising score measurements or were parents of progeny samples with bruising score measurements. Genotyping was performed by Rapid Genomics (Gainesville, Florida, USA); further details can be found in Motazedi et al. [57]. Capture-sequencing data were obtained by paired-end Illumina HiSeq 2000 technology for 20,035 baits. The baits were designed to target the exonic regions of random genes selected to reflect the gene density in the corresponding genomic region. The resulting 100bp paired-end reads were processed as follows. They were assessed for quality control using FastQC v0.11.2 [58]. FastQC Screen v0.5.2 [59] was used to check for contamination. Trimming and filtering was performed with Trimmomatic v0.36 [60]. The paired reads were aligned to the reference genome PGSC-DM v4.03 [61, 62] using BWAmem v0.7.15 [63], then sorted and converted to bam files using SAMtools v1.3.1 [64]. Picard-tools v2.10.1 [65] was used to add group information to the reads. Variant calling was performed with FreeBayes [66].

Variant data preprocessing was performed with the Python library scikit-allel [67]. Samples were filtered out if they had missing values (i.e. no reads) for more than 10% of the variants, leading to retaining 176 samples. Only heterozygous biallelic SNPs were retained for the analysis. Altogether, variants were filtered out if they did not meet each of the following conditions: (i) less than 10% of samples with missing genotype (i.e. no reads), (ii) QualByDepth (QD) score of two or more, and (iii) minor allele frequency larger than 0.01. The QD score of each variant was computed as the quality score of the variant divided by the sum of its coverage for non-homozygous samples. This led to retaining 454,247 SNPs.

Genotype calling was performed using the R package polyRAD [68], using the IteratePopStructLD function. This function iteratively estimates genotype posterior probabilities for each variant/sample pair, using a PCA to estimate samples’ allele frequency based on population structure, and updating genotypes prior probabilities according to the genotype of linked markers. Upon examination of diagnostic plots to assess the fit of observed over expected read depth ratio, the overdispersion parameter was set to 8. The weighted mean genotypes as well as the most probable genotypes were obtained and used in subsequent analyses. Variants were then filtered based on linkage disequilibrium using a custom R script, based on the SNPRelate package [69]. Briefly, for each chromosome, the $r^2$ value between each pair of variants was computed as the squared correlation between complete observations (i.e. excluding missing values). Variants in the chromosome were sorted according to their genomic position, and a random variant was selected as a starting point. The starting variant was added to the set *S* of retained variants. Iteratively, each variant *j* on the right of the starting variant was considered as follows. If there existed a variant *k* within *S* (i.e. the set of retained variants), such that variants *j* and *k* were within 100 kb of each other and the $r^2$ value between *j* and *k* was above 0.5, then *j* was not retained. If no such variant *k* existed, then instead *j* was added to *S*. Once the end of the chromosome was reached, the procedure was continued, starting from the variant on the left of the starting variant, to the beginning of the chromosome. This led to retain 72,847 variants. The value of 0.5 as threshold on the $r^2$ for a pair of marker was selected to only remove markers with redundant information while retaining the richness of the dataset.

#### Population structure analysis

A PCA was performed on the most probable genotypes of the 176 samples, using the R package adegenet [70]. In addition, population structure amongst the samples was investigated using STRUCTURE [71] and DAPC [72]. Briefly, STRUCTURE is a Bayesian model-based clustering method that investigates the population structure amongst a set of samples using multilocus genotype data. DAPC is a multivariate approach that clusters samples in different groups and seeks a low dimensional space that maximises the variance between groups while minimising the variance within groups.

For computational efficiency, 10,000 variants were randomly sampled and used to infer population structure using STRUCTURE. This number of SNPs is amply sufficient to detect population structure accurately [73]. The most probable genotype of the retained variants was used as the input. The number of subpopulations was estimated by running STRUCTURE with a number of subpopulations *nS* ranging from 1 to 10, using the admixture model (samples can be an admixture of the subpopulations) and the correlated allele frequency model (assumes similar allele frequencies across the subpopulations), with a burn-in length of 10,000 and a run length of 20,000. Each run for a particular value of *nS* was replicated five times, using the R package ParallelStructure [74]. The optimal value of $nS = 4$ was selected by inspection of the $\Delta nS$ values [75], which represent the ratio of the average over the five runs of the second derivative of the likelihood (with respect to *nS*) over the likelihood variance. To correct for the effect of label switching, i.e. the fact that in different runs of STRUCTURE with identical parameters, the uncovered subpopulations are not assigned the same label, the correlation between the computed samples’ posterior membership probabilities of the five runs of STRUCTURE (with the optimal value of *nS*) was used to cluster the subpopulations discovered with each run. The final posterior membership probabilities of each sample (i.e. the estimated probability that the sample belongs to each of the *nS* inferred subpopulations) was computed as the mean of the estimated posterior membership probabilities across the five runs.

The DAPC analysis was performed using the R package adegenet [70]. First, a PCA was performed over the samples’ weighted mean genotypes, and the principal components were used to perform a k-means clustering of the samples. The detected clusters correspond to the different subpopulations detected by the algorithm amongst the investigated population. In order to estimate the optimal number of clusters amongst the samples, the k-means clustering was repeated for a number of clusters *nC* ranging from 1 to 10, and the optimal number of clusters was selected as the smallest value of *nC* yielding the lowest BIC score. This led to setting $nC = 5$. Second, a discriminant analysis was performed on the principal components obtained with the PCA. The aim of the discriminant analysis is to find a low-dimensional space that minimises the variance within cluster while maximising the variance between clusters. This space is described by a small number of variables (axes of the space) that are created as linear combinations of the markers. In order to avoid overfitting in the discriminant analysis, only a subset of the principal components were retained for the discriminant analysis. We note that overfitting is not an issue for the clustering part of the analysis, as it is about detecting the optimal number of subpopulations using all genetic information available. The optimal number of principal components to retain was estimated by cross-validation: 90% of the samples were randomly assigned to a training set, and the discriminant analysis was performed on the training set. The remaining 10% of the samples (validation set) were used to estimate the performance of the resulting model in assigning the samples to their respective cluster. The cross-validation scheme was repeated with a different number of principal components used for the discriminant analysis, and the optimal number of principal components to retain was estimated as the highest value for which the performance was still high, and for which the Root Mean Squared Error (RMSE) was still low. A measure similar to the posterior membership probability of samples was computed, which reflects the probability of each sample to be assigned to each of the k clusters given its estimated coordinates in the discriminant analysis space.

#### Genome-wide association study

Phenotypic values for all genotypes were computed using the following linear mixed model:

$$\begin{aligned} y_{ijk} = \mu + G_i + \beta _{(i)j} + \epsilon _{ijk} \end{aligned}$$

where $y_{ijk}$ is the bruising score for tuber *k* of replicate *j* for genotype *i*; $G_i$ is the genotype effect, treated as a fixed effect; $\beta _{(i)j}$ is the biological replicate effect, nested within the genotype effect and treated as a random effect; $\epsilon _{ijk}$ is the residual effect. The Best Linear Unbiased Estimates (BLUEs) obtained were used as the phenotypic value for the GWAS analysis. The model was fitted with the lme4 package in R.

The association analysis was performed using the GWASpoly package [16]. GWASpoly models the effect of the SNPs on the phenotype using the following linear mixed model:

$$\begin{aligned} \textbf{y} = \textbf{X}\varvec{\beta } + \textbf{S}\varvec{\tau } + \textbf{Qv} + \textbf{Zu} + \epsilon \end{aligned}$$

where $\textbf{y}$ is a $n\times 1$ vector of measured phenotypic values; $\varvec{\beta }$ is a $p \times 1$ vector of covariate effects (if any), with $\textbf{X}$ the $n \times p$ covariate incidence matrix; $\varvec{\tau }$ is a $s \times 1$ vector of SNP effects, with *S* is the $n \times s$ incidence matrix; $\textbf{v}$ is a $q \times 1$ vector of subpopulation effects, with $\textbf{Q}$ the $n \times q$ incidence matrix relating the samples to the subpopulations (in practice $\textbf{Q}$ is the matrix of samples posterior membership for the different subpopulations); $\textbf{u}$ is a $n \times 1$ vector of random polygenic effects with variance $Var(\textbf{u}) = \sigma ^2_g\textbf{K}$, where $\sigma ^2_g$ is the genetic variance and $\textbf{K}$ the $n \times n$ kinship matrix (i.e. matrix describing the relationship between samples), with $\textbf{Z}$ the $n \times n$ genotype/phenotype incidence matrix; and $\epsilon$ is a $n \times 1$ vector of residuals, with variance $Var(\epsilon ) = \textbf{I}\sigma ^2_e$, where $\sigma ^2_e$ corresponds to the residual variance. An *F*-test is used to compute a *p*-value for each marker, with the null hypothesis that all SNPs effects (i.e. all values in the vector $\varvec{\tau }$ of SNP effects) are equal to zero. The negative log10 of such *p*-value is thereafter referred to as the marker score. The phenotypic values were normalised for the analysis using the bestNormalize R package [76], which selected the standardized Yeo-Johnson transformation.

Eight possible genetic models that describe the impact of a marker’s dosage on the phenotype were considered, namely:

- the general model: each dosage can have a different arbitrary impact on the phenotype;
- the additive model: the effect of the marker on the phenotype changes linearly with the marker’s dosage;
- the simplex reference dominant model, denoted as 1-dom-ref: the effect of the marker on the phenotype is determined by the presence of at least one copy of the reference allele;
- the simplex alternate dominant model, denoted as 1-dom-alt: the effect of the marker on the phenotype is determined by the presence of at least one copy of the alternate allele;
- the duplex reference dominant model, denoted as 2-dom-ref: the effect of the marker on the phenotype is determined by the presence of at least two copies of the reference allele;
- the duplex alternate dominant model, denoted as 2-dom-alt: the effect of the marker on the phenotype is determined by the presence of at least two copies of the alternate allele;
- the diploidised general model, denoted as diplo-general: all heterozygotes have the same effect, with no constraint on this effect;
- the diploidised additive model, denoted as diplo-additive: all heterozygotes have the same effect, and this effect is halfway between the effect of the two homozygotes.

In addition, similarly to Sharma et al. [18], different population settings that correct for population structure and/or individual relatedness were evaluated:

- Naive model: no correction for population structure nor individual relatedness (kinship matrix $\textbf{K}$ set to a matrix of zeros with diagonal elements set to one), and no covariates added in the model;
- K-only model: accounts for individual relatedness only by computing the kinship matrix as the realised relationship matrix $\textbf{K} = \textbf{MM}^T$ [77], where $\textbf{M}$ is the genotype matrix of variants dosage. The leave-one-chromosome-out method [78] was used, which computes a different kinship matrix for each chromosome. No covariate is added in the model;
- Q only model: accounts for population structure only by adding the samples posterior membership probabilities for the uncovered subpopulations as covariates in the analysis. The kinship matrix $\textbf{K}$ is set to a matrix of zeros with diagonal elements set to one;
- K+Q model: accounts for both individual relatedness and population structure as described above.

In order to correct for population structure, the samples posterior membership probabilities computed with STRUCTURE and DAPC were used, which gave six different population settings: naive model, K model, $\text {Q}_\text {STRUCT}$, $\text {Q}_\text {DAPC}$, $\text {K+Q}_\text {STRUCT}$ and $\text {K+Q}_\text {DAPC}$. This yielded a total of 48 different GWAS settings (eight genetic models $\times$ six population settings).

For each GWAS setting, the ability of the model to control for false positive and false negative was evaluated by computing its inflation factor, which quantifies the deviation of the estimated marker scores from their expected values. For each setting, the inflation factor was computed as the regression coefficient of the markers’ observed scores over their expected scores under the null hypothesis. The best population setting was selected as follows. First, the average inflation score across all genetic models was obtained for each population setting. Second, the population setting yielding the average inflation factor closest to 1 and above 1 was selected. Only the GWAS results for this population setting were considered for the remaining analyses (all genetic models considered).

To correct for multiple testing, a FDR correction was applied with a type-I error level of 0.05. Markers were then retained as significant QTLs only if their computed score was above the significance threshold computed with the FDR method. In addition, any marker whose unadjusted *p*-value was above $10^{-4}$ was retained as a high-scoring marker.

### Transcriptomics

#### Data collection and preprocessing

Transcriptomics data were obtained for 100 progeny samples from crosses with Crop52. Two hours after application of the bruising treatment, samples were taken from one bruised side of each tuber, and the samples obtained from the three tubers of a biological replicate were pooled and snap-frozen, ground, and stored at -80$^\circ$C. One biological replicate for each genotype was chosen for RNA measurement. Transcriptomics measurements were obtained for 100 samples using an Illumina NovaSeq 6000 platform (Illumina, San Diego, USA), with a Lexogen SENSE mRNA polyA library.

The resulting reads were processed as follows. Read quality was assessed with FastQC v0.11.7 [58], and rRNA contaminants were removed using SortMeRNA [79] using default parameters. Trimming was performed with BBtools v37.93 BBDuk [80], with the flag forcetrimleft set to 15, trimpolyg to 30, trimpoly to 30, k to 13, qtrim to r, trimq to 10 and minlength to 50. The reads were then aligned to the reference genome PGSC-DM (genome assembly version v4.03 and gene annotation v4.03) [61, 62] and the number of reads overlapping each gene in the reference genome was computed using STAR v2.6.1 [81] with the following flag settings: alignMatesGapMax to 20000, outQSconversionAdd to -31, outFilterScoreMinOverLread to 0, outFilterMatchNminOverLread to 0, outFilterMatchNmin to 40, alignSJDBoverhangMin to 10, alignIntronMax to 200000, quantMode to “GeneCounts".

The remaining data processing was performed with R. Transcribed genes for which at least five samples had read counts lower than 5 were filtered out; 25,163 transcribed genes were retained. The BiomaRt package [82] was used to retrieve the description of the genes and associated GO annotations from their ensembl ID.

#### Differential expression analysis

As the RNA measurements were done on one biological replicate for each genotype, the mean bruising score over all three tubers for the corresponding biological replicate was used for the differential expression analysis. Samples with a mean bruising score of 1 or less were classified in the “low bruising” group. Samples with a mean bruising score of 2 or more were classified in the “high bruising” group. The remaining samples were discarded from the analysis. This yielded a low bruising group of 41 samples, and a high bruising group of 33 samples. The DESeq2 package [83] was used to perform the differential expression analysis between the low and high bruising groups, with the untransformed read counts of the transcribed genes as input. The DESeq2 package normalises the read counts via the Variance Stabilising Transformation method, and then tests for each gene the null hypothesis that the expression strength of the gene is identical between the two bruising groups using a Wald test on the log fold-change of the gene. The transcribed genes’ *p*-values obtained were corrected for multiple testing using the FDR correction (adjustment performed by the DESeq2package). Transcribed genes were considered as significantly differentially expressed if their FDR-adjusted *p*-value was below 0.05.

Enrichment of the molecular function- and biological process-related GO categories for differentially expressed genes was computed with the gage R package [84]. gage assesses whether a given set of genes (in this case, a set of genes grouped in the same GO category) is enriched for differentially expressed genes by comparing its mean gene score to the mean gene score of all the genes not in the set (referred to as the background set). Here, the differential expression score of the genes, i.e. -log10 of their adjusted *p*-value, was used as the gene scores. The comparison is done using a prototype two-sample t-test, which contrasts the set of genes of interest to a virtual random set of genes from the background set of the same size. The resulting set of *p*-values were corrected for multiple testing via the FDR correction. GO terms were detected as significantly enriched for differentially expressed genes if the corresponding adjusted *p*-value was below 0.05.

When comparing findings from the literature with differentially expressed genes, similarity between gene sequences was computed using the BLASTN tool from the NCBI website [85], and limiting the results to “Solanum tuberosum (taxid:4113)” (all other parameters were left to their default settings).

## Supplementary Information

**Additional file 1.** Supplementary tables and figures.

**Additional file 2.** Supplementary Table 3.

## Data Availability

The genomics data reported in this paper have been deposited in the NCBI Sequence Read Archive under the BioProject number PRJNA856716 (https://www.ncbi.nlm.nih.gov/sra/PRJNA856716), with BioSample accessions SAMN29585022 to SAMN29585199. The transcriptomics data (raw and processed files) have been deposited in NCBI’s Gene Expression Omnibus [86] with accession number GSE208439 (https://www.ncbi.nlm.nih.gov/geo/query/acc.cgi?acc=GSE208439), with Sample accessions GSM6346379 to GSM6346478. The code used for the analyses is available on GitHub at https://github.com/PlantandFoodResearch/Tuber_bruising.

## Abbreviations

ABA: abscisic acid
DAPC: Discriminant analysis of principal components
DE: differentially expressed
FDR: false discovery rate
GWAS: genome-wide expression analysis
LD: linkage disequilibrium
PCA: principal components analysis
PPO: polyphenol oxidase
QTL: quantitative trait loci
SNP: single nucleotide polymorphism

## References

[1] FAOSTAT. FAO Statistical Yearbook - World Food and Agriculture 2021; Figure 21. 2021. 10.1016/j.cell.2016.08.029.

[2] Werij JS, Kloosterman B, Celis-Gamboa C, De Vos CHR, America T, Visser RGF (2007). Unravelling enzymatic discoloration in potato through a combined approach of candidate genes, QTL, and expression analysis. Theor Appl Genet..

[3] Kloosterman B, Abelenda JA, Gomez MDMC, Oortwijn M, De Boer JM, Kowitwanich K (2013). Naturally occurring allele diversity allows potato cultivation in northern latitudes. Nature..

[4] Bisognin DA, Manrique-Carpintero NC, Douches DS (2018). QTL Analysis of Tuber Dormancy and Sprouting in Potato. Am J Potato Res..

[5] Hara-Skrzypiec A, Śliwka J, Jakuczun H, Zimnoch-Guzowska E (2018). Quantitative trait loci for tuber blackspot bruise and enzymatic discoloration susceptibility in diploid potato. Mol Gen Genomics..

[6] Bradshaw JE, Hackett CA, Pande B, Waugh R, Bryan GJ (2008). QTL mapping of yield, agronomic and quality traits in tetraploid potato (Solanum tuberosum subsp. tuberosum). Theor Appl Genet.

[7] Malosetti M, Van Der Linden CG, Vosman B, Van Eeuwijk FA (2007). A mixed-model approach to association mapping using pedigree information with an illustration of resistance to Phytophthora infestans in potato. Genetics..

[8] Li L, Paulo MJ, Strahwald J, Lübeck J, Hofferbert HR, Tacke E (2008). Natural DNA variation at candidate loci is associated with potato chip color, tuber starch content, yield and starch yield. Theor Appl Genet..

[9] Urbany C, Stich B, Schmidt L, Simon L, Berding H, Junghans H (2011). Association genetics in Solanum tuberosum provides new insights into potato tuber bruising and enzymatic tissue discoloration. BMC Genomics..

[10] Fischer M, Schreiber L, Colby T, Kuckenberg M, Tacke E, Hofferbert HR (2013). Novel candidate genes influencing natural variation in potato tuber cold sweetening identified by comparative proteomics and association mapping. BMC Plant Biol..

[11] Schreiber L, Nader-Nieto AC, Schönhals EM, Walkemeier B, Gebhardt C (2014). SNPs in genes functional in starch-sugar interconversion associate with natural variation of tuber starch and sugar content of potato (Solanum tuberosum L.). G3 Genes Genomes Genet.

[12] Schönhals EM, Ortega F, Barandalla L, Aragones A, Ruiz de Galarreta JI, Liao JC (2016). Identification and reproducibility of diagnostic DNA markers for tuber starch and yield optimization in a novel association mapping population of potato (Solanum tuberosum L.). Theor Appl Genet.

[13] Baldwin SJ, Dodds KG, Auvray B, Genet RA, Macknight RC, Jacobs JME (2011). Association mapping of cold-induced sweetening in potato using historical phenotypic data. Ann Appl Biol..

[14] Carpenter MA, Joyce NI, Genet RA, Cooper RD, Murray SR, Noble AD (2015). Starch phosphorylation in potato tubers is influenced by allelic variation in the genes encoding glucan water dikinase, starch branching enzymes I and II, and starch synthase III. Front Plant Sci.

[15] D’hoop BB, Keizer PLC, Paulo MJ, Visser RGF, van Eeuwijk FA, van Eck HJ (2014). Identification of agronomically important QTL in tetraploid potato cultivars using a marker-trait association analysis. Theor Appl Genet.

[16] Rosyara UR, De Jong WS, Douches DS, Endelman JB. Software for Genome-Wide Association Studies in Autopolyploids and Its Application to Potato. Plant Genome. 2016;9(2). 10.3835/plantgenome2015.08.0073. 10.3835/plantgenome2015.08.0073 27898814

[17] Schönhals EM, Ding J, Ritter E, Paulo MJ, Cara N, Tacke E (2017). Physical mapping of QTL for tuber yield, starch content and starch yield in tetraploid potato (Solanum tuberosum L.) by means of genome wide genotyping by sequencing and the 8.3 K SolCAP SNP array. BMC Genomics.

[18] Sharma SK, MacKenzie K, McLean K, Dale F, Daniels S, Bryan GJ (2018). Linkage disequilibrium and evaluation of genome-wide association mapping models in tetraploid potato. G3 Genes Genomes Genet.

[19] Michaelson JJ, Loguercio S, Beyer A. Detection and interpretation of expression quantitative trait loci (eQTL). Methods. 2009. 10.1016/j.ymeth.2009.03.004. 10.1016/j.ymeth.2009.03.004 19303049

[20] Bazakos C, Hanemian M, Trontin C, Jiménez-Gómez JM, Loudet O (2017). New Strategies and Tools in Quantitative Genetics: How to Go from the Phenotype to the Genotype. Ann Rev Plant Biol..

[21] Liu Y, Lin-Wang K, Deng C, Warran B, Wang L, Yu B (2015). Comparative transcriptome analysis of white and purple potato to identify genes involved in anthocyanin biosynthesis. PloS ONE..

[22] Aliche EB, Gengler T, Hoendervangers I, Oortwijn M, Bachem CW, Borm T (2022). Transcriptomic Responses of Potato to Drought Stress. Potato Res..

[23] Saidi A, Hajibarat Z (2020). Application of Next Generation Sequencing, GWAS, RNA seq, WGRS, for genetic improvement of potato (Solanum tuberosum L.) under drought stress. Biocatalysis Agric Biotechnol.

[24] Lin Q, Xie Y, Guan W, Duan Y, Wang Z, Sun C (2019). Combined transcriptomic and proteomic analysis of cold stress induced sugar accumulation and heat shock proteins expression during postharvest potato tuber storage. Food Chem..

[25] Gao L, Tu ZJ, Millett BP, Bradeen JM (2013). Insights into organ-specific pathogen defense responses in plants: RNA-seq analysis of potato tuber-Phytophthora infestans interactions. BMC Genomics..

[26] Li Q, Qin Y, Hu X, Li G, Ding H, Xiong X (2020). Transcriptome analysis uncovers the gene expression profile of salt-stressed potato (Solanum tuberosum L.). Sci Rep.

[27] Alexandersson E, Kushwaha S, Subedi A, Weighill D, Climer S, Jacobson D (2020). Linking crop traits to transcriptome differences in a progeny population of tetraploid potato. BMC Plant Biol..

[28] Boutsika A, Tanou G, Xanthopoulou A, Samiotaki M, Nianiou-Obeidat I, Ganopoulos I (2022). Insights and advances in integrating multi-omic approaches for potato crop improvement. Sci Hortic..

[29] Tiwari JK, Buckseth T, Zinta R, Bhatia N, Dalamu D, Naik S (2022). Germplasm, Breeding, and Genomics in Potato Improvement of Biotic and Abiotic Stresses Tolerance. Front Plant Sci..

[30] Ramšak Ž, Petek M, Baebler Š, Dobnik D, Gruden K, Ramšak Ž, Coll A (2021). RNA Sequencing Analyses for Deciphering Potato Molecular Responses. Solanum tuberosum: Methods and Protocols.

[31] Caruana BM, Pembleton LW, Constable F, Rodoni B, Slater AT, Cogan NOI (2019). Validation of genotyping by sequencing using transcriptomics for diversity and application of genomic selection in tetraploid potato. Front Plant Sci..

[32] Petek M, Zagorščak M, Ramšak Ž, Sanders S, Tomaž Š, Tseng E (2020). Cultivar-specific transcriptome and pan-transcriptome reconstruction of tetraploid potato. Sci Data..

[33] Wright P, Triggs C, Anderson J (2005). Effects of specific gravity and cultivar on susceptibility of potato (Solanum tuberosum) tubers to blackspot bruising and bacterial soft rot. N Z J Crop Hortic Sci..

[34] Goyer A, Pellé J (2018). Relationships between tyrosine, phenylalanine, chlorogenic acid, and ascorbic acid concentrations and blackspot biochemical potential and blackspot susceptibility in stored russet potatoes. J Sci Food Agric..

[35] Storey M, Vreugdenhil D, Bradshaw J, Gebhardt C, Govers F, Mackerron DKL, Taylor MA (2007). The harvested crop. Potato Biology and Biotechnology.

[36] Urbany C, Colby T, Stich B, Schmidt L, Schmidt J, Gebhardt C (2012). Analysis of Natural Variation of the Potato Tuber Proteome Reveals Novel Candidate Genes for Tuber Bruising. J Proteome Res..

[37] Van Berloo R, Hutten R, Van Eck H, Visser R (2007). An online potato pedigree database resource. Potato Res..

[38] Zhu JK (2016). Abiotic stress signaling and responses in plants. Cell..

[39] Blanco FA, Zanetti ME, Casalongué CA, Daleo GR (2006). Molecular characterization of a potato MAP kinase transcriptionally regulated by multiple environmental stresses. Plant Physiol Biochem..

[40] Müller M, Munné-Bosch S (2015). Ethylene response factors: a key regulatory hub in hormone and stress signaling. Plant Physiol..

[41] Charfeddine M, Saïdi MN, Charfeddine S, Hammami A, Gargouri Bouzid R (2015). Genome-wide analysis and expression profiling of the ERF transcription factor family in potato (Solanum tuberosum L.). Mol Biotechnol.

[42] Sano T, Nagata T (2002). The possible involvement of a phosphate-induced transcription factor encoded by Phi-2 gene from tobacco in ABA-signaling pathways. Plant Cell Physiol..

[43] Sprenger H, Kurowsky C, Horn R, Erban A, Seddig S, Rudack K (2016). The drought response of potato reference cultivars with contrasting tolerance. Plant Cell Environ..

[44] Perl A, Shaul O, Galili G (1992). Regulation of lysine synthesis in transgenic potato plants expressing a bacterial dihydrodipicolinate synthase in their chloroplasts. Plant Mol Biol..

[45] Wu D, He G, Tian W, Saleem M, Li D, Huang Y (2021). OPT gene family analysis of potato (Solanum tuberosum) responding to heavy metal stress: Comparative omics and co-expression networks revealed the underlying core templates and specific response patterns. Int J Biol Macromol..

[46] Hamilton ES, Schlegel AM, Haswell ES (2015). United in diversity: mechanosensitive ion channels in plants. Annu Rev Plant Biol..

[47] Hamant O, Haswell ES (2017). Life behind the wall: sensing mechanical cues in plants. BMC Biol..

[48] Van der Does D, Boutrot F, Engelsdorf T, Rhodes J, McKenna JF, Vernhettes S (2017). The Arabidopsis leucine-rich repeat receptor kinase MIK2/LRR-KISS connects cell wall integrity sensing, root growth and response to abiotic and biotic stresses. PLoS Genet..

[49] Rowland O, Ludwig AA, Merrick CJ, Baillieul F, Tracy FE, Durrant WE (2005). Functional analysis of Avr9/Cf-9 rapidly elicited genes identifies a protein kinase, ACIK1, that is essential for full Cf-9-dependent disease resistance in tomato. Plant Cell..

[50] Jupe F, Pritchard L, Etherington GJ, MacKenzie K, Cock PJ, Wright F (2012). Identification and localisation of the NB-LRR gene family within the potato genome. BMC Genomics..

[51] Bacete L, Melida H, Miedes E, Molina A (2018). Plant cell wall-mediated immunity: cell wall changes trigger disease resistance responses. Plant J..

[52] Heibges A, Glaczinski H, Ballvora A, Salamini F, Gebhardt C. Structural diversity and organization of three gene families for Kunitz-type enzyme inhibitors from potato tubers (Solanum tuberosum L.). Mol Genet Genomics. 2003;269(4):526–534. 10.1007/s00438-003-0860-0. 10.1007/s00438-003-0860-0 12783302

[53] Odeny DA, Stich B, Gebhardt C (2010). Physical organization of mixed protease inhibitor gene clusters, coordinated expression and association with resistance to late blight at the StKI locus on potato chromosome III. Plant Cell Environ..

[54] Singh B, Bhardwaj V, Kaur K, Kukreja S, Goutam U (2021). Potato periderm is the first layer of defence against biotic and abiotic stresses: a review. Potato Res..

[55] Wang M, Zhu X, Wang K, Lu C, Luo M, Shan T (2018). A wheat caffeic acid 3-O-methyltransferase TaCOMT-3D positively contributes to both resistance to sharp eyespot disease and stem mechanical strength. Sci Rep..

[56] Shepherd LVT, Alexander CJ, Hackett CA, McRae D, Sungurtas JA, Verrall SR (2015). Impacts on the metabolome of down-regulating polyphenol oxidase in potato tubers. Transgenic Res..

[57] Motazedi E, de Ridder D, Finkers R, Baldwin S, Thomson S, Monaghan K (2018). TriPoly: haplotype estimation for polyploids using sequencing data of related individuals. Bioinformatics..

[58] Andrews S, Krueger F, Segonds-Pichon A, Biggins L, Krueger C, Wingett S. FastQC: a quality control tool for high throughput sequence data. 2018. https://www.bioinformatics.babraham.ac.uk/projects/fastqc/ Accessed 04 May 2023.

[59] Wingett SW, Andrews S (2018). FastQ Screen: A tool for multi-genome mapping and quality control. F1000 Research.

[60] Bolger AM, Lohse M, Usadel B (2014). Trimmomatic: a flexible trimmer for Illumina sequence data. Bioinformatics..

[61] Sharma SK, Bolser D, de Boer J, Sønderkær M, Amoros W, Carboni MF (2013). Construction of reference chromosome-scale pseudomolecules for potato: Integrating the potato genome with genetic and physical maps. G3 Genes Genomes Genet.

[62] Xu X, Pan S, Cheng S, Zhang B, Mu D, Ni P (2011). Genome sequence and analysis of the tuber crop potato. Nature..

[63] Li H. Aligning sequence reads, clone sequences and assembly contigs with BWA-MEM. arXiv preprint arXiv:1303.3997. 2013. 10.48550/arXiv.1303.3997.

[64] Li H, Handsaker B, Wysoker A, Fennell T, Ruan J, Homer N (2009). The Sequence Alignment/Map format and SAMtools. Bioinformatics..

[65] Picard toolkit. Broad Institute. 2019. http://broadinstitute.github.io/picard/. Accessed 04 May 2023.

[66] Garrison E, Marth G. Haplotype-based variant detection from short-read sequencing. arXiv preprint arXiv:1207.3907. 2012. 10.48550/arXiv.1207.3907

[67] Miles A, pyup io Bot, R M, Ralph P, Harding N, Pisupati R, et al. cggh/scikit-allel: v1.3.2. 2020. 10.5281/ZENODO.3976233.

[68] Clark LV, Lipka AE, Sacks EJ (2019). polyRAD: Genotype calling with uncertainty from sequencing data in polyploids and diploids. G3 Genes Genomes Genet.

[69] Zheng X, Levine D, Shen J, Gogarten SM, Laurie C, Weir BS (2012). A high-performance computing toolset for relatedness and principal component analysis of SNP data. Bioinformatics..

[70] Jombart T, Ahmed I (2011). adegenet 1.3-1: New tools for the analysis of genome-wide SNP data. Bioinformatics.

[71] Pritchard JK, Stephens M, Donnelly P (2000). Inference of population structure using multilocus genotype data. Genetics..

[72] Jombart T, Devillard S, Balloux F (2010). Discriminant analysis of principal components: A new method for the analysis of genetically structured populations. BMC Genet..

[73] Turakulov R, Easteal S (2003). Number of SNPS loci needed to detect population structure. Hum Hered..

[74] Besnier F, Glover KA (2013). ParallelStructure: A R Package to Distribute Parallel Runs of the Population Genetics Program STRUCTURE on Multi-Core Computers. PLoS ONE..

[75] Evanno G, Regnaut S, Goudet J (2005). Detecting the number of clusters of individuals using the software STRUCTURE: A simulation study. Mol Ecol..

[76] Peterson RA (2021). Finding Optimal Normalizing Transformations via bestNormalize. R J.

[77] VanRaden PM (2008). Efficient methods to compute genomic predictions. J Dairy Sci..

[78] Yang J, Zaitlen NA, Goddard ME, Visscher PM, Price AL (2014). Advantages and pitfalls in the application of mixed-model association methods. Nat Genet..

[79] Kopylova E, Noé L, Touzet H (2012). SortMeRNA: Fast and accurate filtering of ribosomal RNAs in metatranscriptomic data. Bioinformatics..

[80] Bushnell B. BBMap short read aligner, and other bioinformatic tools. 2016. https://sourceforge.net/projects/bbmap/. Accessed 04 May 2023.

[81] Dobin A, Davis CA, Schlesinger F, Drenkow J, Zaleski C, Jha S (2013). STAR: Ultrafast universal RNA-seq aligner. Bioinformatics..

[82] Durinck S, Spellman PT, Birney E, Huber W (2009). Mapping identifiers for the integration of genomic datasets with the R/ Bioconductor package biomaRt. Nat Protoc..

[83] Love MI, Huber W, Anders S (2014). Moderated estimation of fold change and dispersion for RNA-seq data with DESeq2. Genome Biol..

[84] Luo W, Friedman MS, Shedden K, Hankenson KD, Woolf PJ (2009). GAGE: Generally applicable gene set enrichment for pathway analysis. BMC Bioinformatics..

[85] Zhang Z, Schwartz S, Wagner L, Miller W (2000). A greedy algorithm for aligning DNA sequences. J Comput Biol..

[86] Edgar R, Domrachev M, Lash AE (2002). Gene Expression Omnibus: NCBI gene expression and hybridization array data repository. Nucleic Acids Res..

