# Supplementary material for: Investigating the genetic components of tuber bruising in a breeding population of tetraploid potatoes
Source: BMC Plant Biol. 2023 May 5;23:238. doi: 10.1186/s12870-023-04255-2 (PMC10161554; doi:10.1186/s12870-023-04255-2)
Supplement: Supplementary file 1 — Additional file 1. Supplementary tables and figures. [file 12870_2023_4255_MOESM1_ESM.pdf]

Investigating the genetic components of tuber bruising in a breeding population of tetraploid potatoes

Supplementary Tables and Figures

O. Angelin-Bonnet, S. Thomson, M. Vignes, P.J. Biggs, K. Monaghan, R. Bloomer, K. Wright, S. Baldwin

Supplementary Table 1: Genomic position of GWAS high-scoring markers (i.e. markers with an unadjusted p-value $< 10^{-4}$). Markers with significant scores are highlighted in bold. For a given marker, only the genetic model yielding the highest score is presented. Marker effects cannot be estimated for general and diplo-general models.

Chromosome	Position (bp)	Genetic model	GWAS score	Estimated effect
ST4.03ch00	22,680,252	general	4.41	*NA*
ST4.03ch01	6,317,643	diplo-general	4.15	*NA*
ST4.03ch01	7,671,100	diplo-general	4.43	*NA*
ST4.03ch01	8,653,747	diplo-general	4.69	*NA*
**ST4.03ch01**	**12,842,648**	**diplo-general**	**6.85**	***NA***
ST4.03ch01	13,334,335	1-dom-alt	5.24	-0.82
ST4.03ch01	13,544,786	additive	4.00	-0.59
ST4.03ch01	42,743,974	diplo-general	4.00	*NA*
ST4.03ch01	42,949,112	additive	4.18	-0.56
ST4.03ch01	52,529,264	diplo-general	4.85	*NA*
ST4.03ch01	67,700,505	2-dom-alt	4.31	-1.15
ST4.03ch02	36,941,161	1-dom-alt	4.59	1.13
ST4.03ch02	41,485,307	additive	4.09	-0.41
ST4.03ch03	52,006,977	additive	4.21	-0.36
ST4.03ch05	1,556,077	additive	5.38	-0.45
ST4.03ch06	47,752,695	2-dom-alt	4.07	-0.83
ST4.03ch06	48,310,651	additive	4.12	0.46
**ST4.03ch07**	**4,990,295**	**1-dom-ref**	**3.97**	**1.00**
ST4.03ch07	7,110,738	additive	4.08	-0.42
ST4.03ch07	50,747,618	2-dom-alt	4.37	-1.02
ST4.03ch08	3,246,504	2-dom-alt	4.12	-0.59
ST4.03ch08	3,419,017	additive	4.02	-0.59
**ST4.03ch08**	**45,864,760**	**2-dom-ref**	**4.12**	**-1.04**
ST4.03ch08	45,873,451	diplo-additive	4.17	-0.53
**ST4.03ch08**	**45,954,553**	**1-dom-ref**	**4.03**	**-1.31**
**ST4.03ch08**	**49,061,782**	**1-dom-ref**	**3.86**	**0.55**
ST4.03ch08	49,372,500	additive	5.15	-0.79
ST4.03ch08	51,252,130	diplo-general	4.30	*NA*
ST4.03ch08	53,167,792	diplo-general	4.17	*NA*
ST4.03ch08	53,220,866	2-dom-alt	4.72	0.64
ST4.03ch08	53,221,167	2-dom-alt	4.61	0.67
ST4.03ch09	11,546,208	2-dom-alt	4.12	0.69
ST4.03ch09	26,418,807	additive	4.01	-0.77
ST4.03ch11	859,574	additive	4.71	0.69
ST4.03ch11	980,896	additive	5.10	0.69
ST4.03ch11	1,103,201	additive	5.26	0.71
ST4.03ch11	1,163,793	additive	4.07	-0.60
ST4.03ch11	3,864,951	additive	4.29	-0.51

Supplementary Table 2: Differentially expressed genes between high- and low-bruising tubers. A positive log2(Fold-change) indicates that the corresponding gene is more expressed in high-bruising tubers than low-bruising tubers.

Chromosome	Position (Mb)	Gene ID	Description	Adjusted p-value	log2(Fold-change)
ST4.03ch00	37.98	PGSC0003DMG400022107	Peptide transporter	0.049	0.99
ST4.03ch01	58.28	PGSC0003DMG400022661	*Conserved gene of unknown function*	0.036	-0.20
ST4.03ch01	81.50	PGSC0003DMG400029752	*Gene of unknown function*	0.049	1.91
ST4.03ch01	84.46	PGSC0003DMG400025889	Phi-2	0.005	0.48
ST4.03ch02	22.62	PGSC0003DMG400003322	*Conserved gene of unknown function*	0.049	0.38
ST4.03ch02	33.23	PGSC0003DMG400031752	Leucine-rich repeat receptor kinase	0.027	0.88
ST4.03ch02	41.35	PGSC0003DMG400026406	*Conserved gene of unknown function*	0.049	0.45
ST4.03ch02	42.35	PGSC0003DMG400021423	Homeodomain leucine-zipper 1	0.031	0.76
ST4.03ch03	0.54	PGSC0003DMG400013449	SNF2 domain-containing protein	0.049	-0.96
ST4.03ch03	9.26	PGSC0003DMG400019758	Dihydrodipicolinate synthase, chloroplastic	0.049	-0.41
ST4.03ch03	34.87	PGSC0003DMG400030266	*Gene of unknown function*	0.049	1.34
ST4.03ch03	43.95	PGSC0003DMG400010129	Aspartic protease inhibitor 10	0.019	-4.26
ST4.03ch03	48.46	PGSC0003DMG400033693	UPA16	0.005	2.59
ST4.03ch03	48.54	PGSC0003DMG400033681	GTP-binding protein alpha subunit, gna	0.049	-0.18
ST4.03ch03	49.37	PGSC0003DMG400010173	Avr9/Cf-9 rapidly elicited protein 140	0.049	-0.43
ST4.03ch03	49.71	PGSC0003DMG400010136	Stigma expressed protein	0.049	-1.70
ST4.03ch03	59.90	PGSC0003DMG400002638	Phospholipase	0.018	3.43
ST4.03ch04	51.60	PGSC0003DMG400020625	AP2/ERF domain-containing transcription factor	0.030	0.44
ST4.03ch04	55.45	PGSC0003DMG401019696	Cell division protein kinase 7	0.049	-0.75
ST4.03ch04	67.80	PGSC0003DMG400008035	*Gene of unknown function*	0.019	-0.75
ST4.03ch04	71.21	PGSC0003DMG400009885	Pentatricopeptide repeat-containing protein	0.049	0.40
ST4.03ch04	71.27	PGSC0003DMG400009888	Mechanosensitive ion channel domain-containing protein	0.049	-0.40
ST4.03ch05	3.47	PGSC0003DMG402030529	*Conserved gene of unknown function*	0.041	1.28
ST4.03ch05	10.35	PGSC0003DMG400018614	*Conserved gene of unknown function*	0.040	0.77
ST4.03ch05	46.05	PGSC0003DMG400014585	Restin	0.026	-2.03
ST4.03ch06	0.66	PGSC0003DMG400014638	Adenosine monophosphate binding protein 1 AMPBP1	0.026	0.78
ST4.03ch06	50.13	PGSC0003DMG400028488	DnaJ protein	0.049	0.70
ST4.03ch06	54.75	PGSC0003DMG400005890	16kDa membrane protein	0.041	2.48
ST4.03ch06	56.08	PGSC0003DMG400006606	Peptide transporter	0.049	1.05
ST4.03ch07	1.26	PGSC0003DMG401011206	Vacuolar ATP synthase subunit h	0.049	-0.23
ST4.03ch07	50.98	PGSC0003DMG400017364	KLTH0D16148p	0.049	0.44
ST4.03ch07	53.82	PGSC0003DMG400019241	*Conserved gene of unknown function*	0.049	0.92
ST4.03ch08	0.39	PGSC0003DMG400012485	Heat shock protein 70 (HSP70)-interacting protein	0.049	-0.57
ST4.03ch08	25.97	PGSC0003DMG400025202	*Gene of unknown function*	0.010	-2.19

Supplementary Table 2: Differentially expressed genes between high- and low-bruising tubers. A positive log2(Fold-change) indicates that the corresponding gene is more expressed in high-bruising tubers than low-bruising tubers. *(continued)*

Chromosome	Position (Mb)	Gene ID	Description	Adjusted p-value	log2(Fold-change)
ST4.03ch08	47.76	PGSC0003DMG400019806	Disease resistance protein RGA3	0.041	-1.73
ST4.03ch08	51.26	PGSC0003DMG400026229	Ubiquitin carboxyl-terminal hydrolase isozyme L3	0.049	0.50
ST4.03ch08	52.57	PGSC0003DMG400004795	Nitrate transporter	0.049	-0.76
ST4.03ch08	53.73	PGSC0003DMG400022746	Anthocyanin synthase	0.049	-1.10
ST4.03ch08	54.68	PGSC0003DMG400012215	DNA binding protein	0.040	-0.26
ST4.03ch08	56.26	PGSC0003DMG400012137	Ring finger protein	0.019	0.96
ST4.03ch09	1.15	PGSC0003DMG400024270	*Conserved gene of unknown function*	0.027	-0.75
ST4.03ch09	7.32	PGSC0003DMG400023931	Pollen allergen Amb a 1.1	0.049	0.77
ST4.03ch09	31.22	PGSC0003DMG400027944	RNA binding protein	0.019	-0.33
ST4.03ch09	43.86	PGSC0003DMG400017655	Phototropic-responsive NPH3 family protein	0.049	0.67
ST4.03ch09	50.74	PGSC0003DMG400003820	Oligopeptide transporter	0.049	-1.43
ST4.03ch09	54.00	PGSC0003DMG400032247	Vicilin	0.033	1.73
ST4.03ch10	32.96	PGSC0003DMG400015948	*Conserved gene of unknown function*	0.005	0.83
ST4.03ch10	52.68	PGSC0003DMG400008596	Cc-nbs-lrr resistance protein	0.049	0.58
ST4.03ch10	58.19	PGSC0003DMG400029856	N-acylneuraminate-9-phosphatase	0.003	0.75
ST4.03ch10	58.51	PGSC0003DMG400008143	RecA protein	0.026	1.06
ST4.03ch11	0.25	PGSC0003DMG400013297	Oligopeptide transporter OPT family	0.041	-0.54
ST4.03ch11	0.67	PGSC0003DMG400013342	Caffeic acid O-methyltransferase II	0.003	-3.42
ST4.03ch11	9.57	PGSC0003DMG400027793	Transmembrane protein 50a	0.049	0.24
ST4.03ch12	39.80	PGSC0003DMG400002452	Big map kinase/bmk	0.049	-0.27
ST4.03ch12	52.73	PGSC0003DMG400004363	Auxin-regulated dual specificity cytosolic kinase	0.049	-0.65
ST4.03ch12	57.62	PGSC0003DMG402029307	Organic anion transporter	0.020	-0.34
ST4.03ch12	59.38	PGSC0003DMG400004667	Rhg4-like receptor kinase II	0.049	-0.59

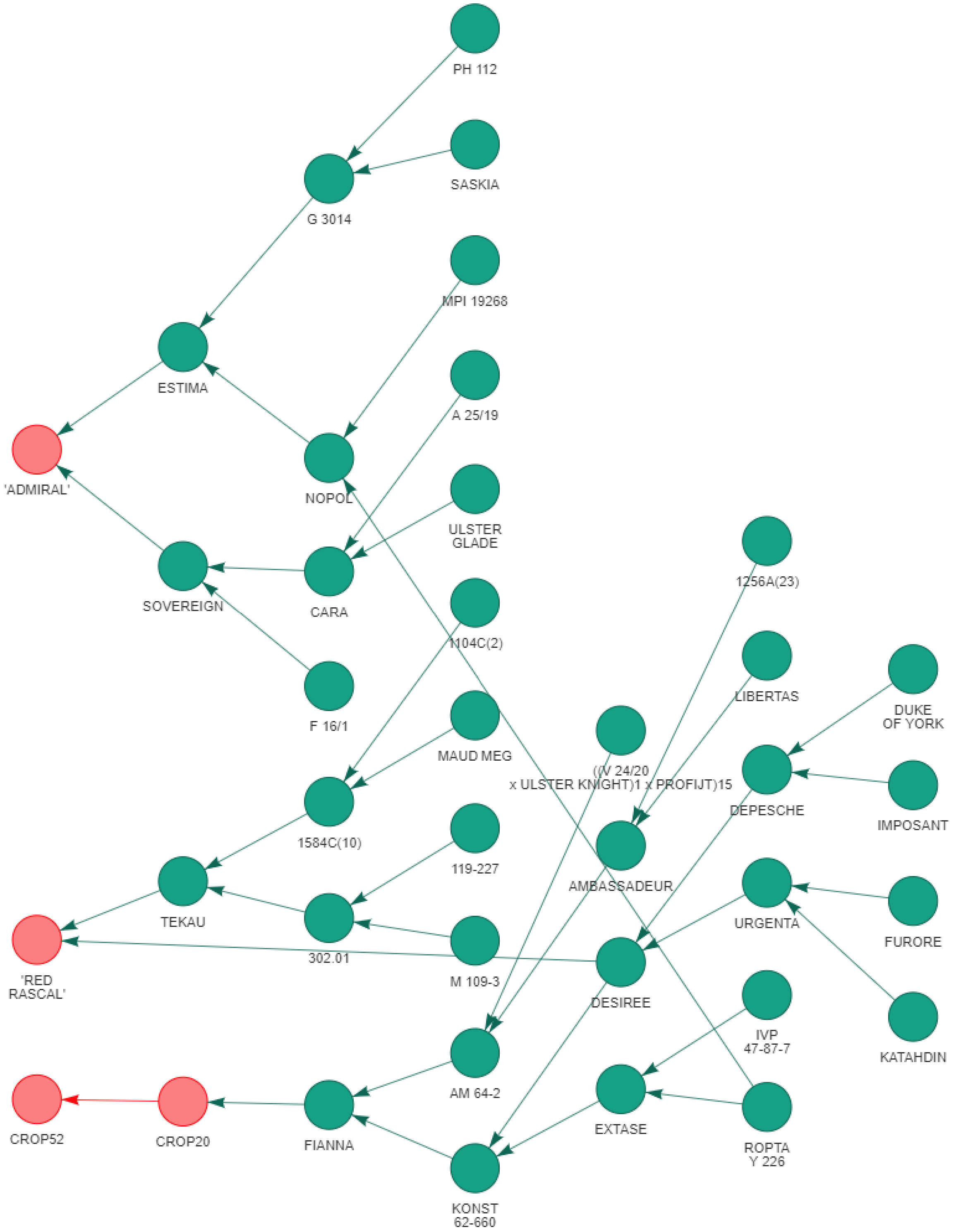

Supplementary Figure 1: Pedigree of ‘Admiral’, ‘Red Rascal’, Crop20 and Crop52 obtained from the Potato Pedigree Database. Red nodes represent the parents used in the crosses for the this study.

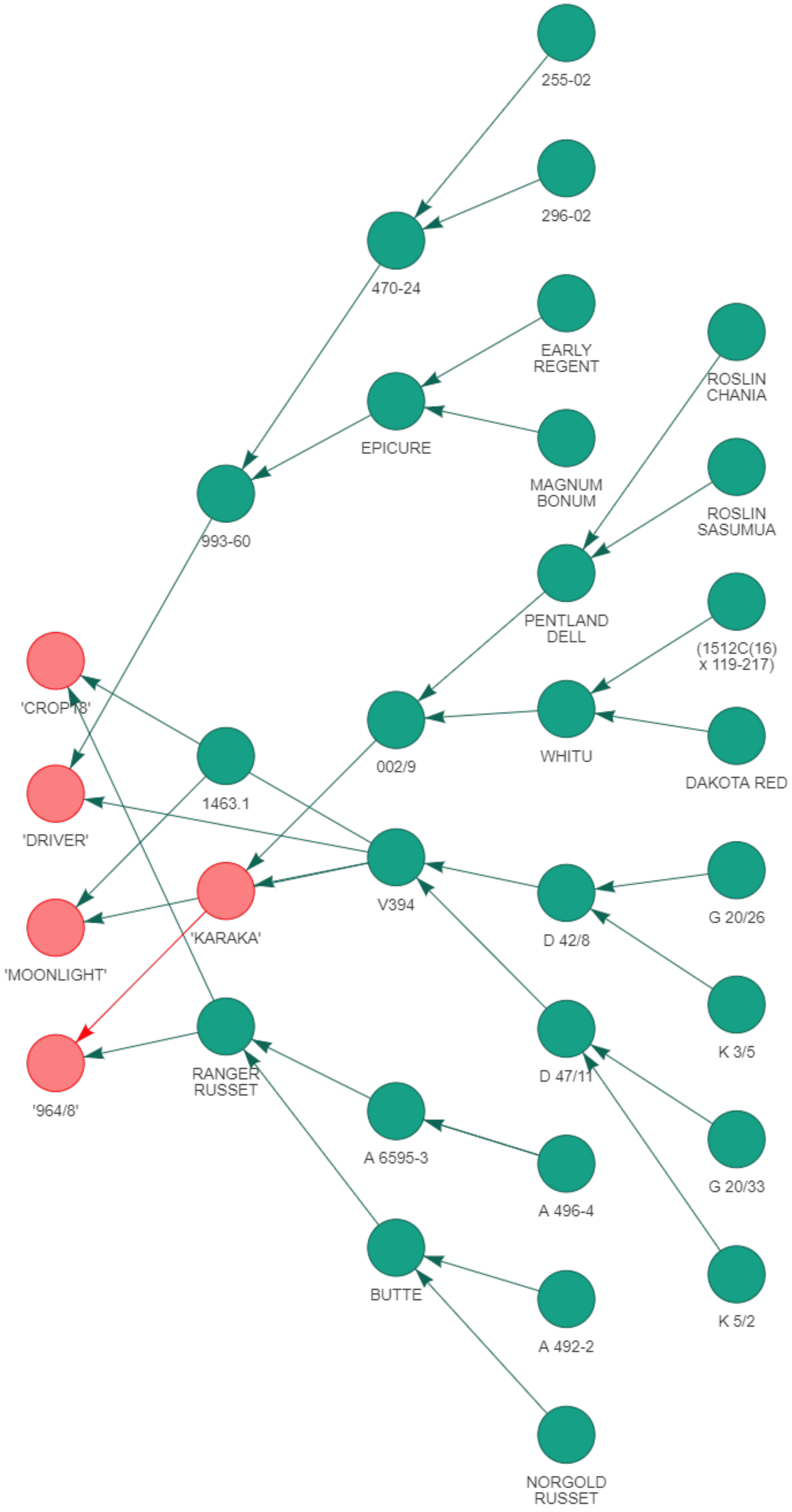

Supplementary Figure 2: Pedigree of ‘Crop18’, ‘Karaka’, ‘Driver’, ‘Moonlight’ and ‘964/8’ obtained from the Potato Pedigree Database. Red nodes represent the parents used in the crosses for the this study.

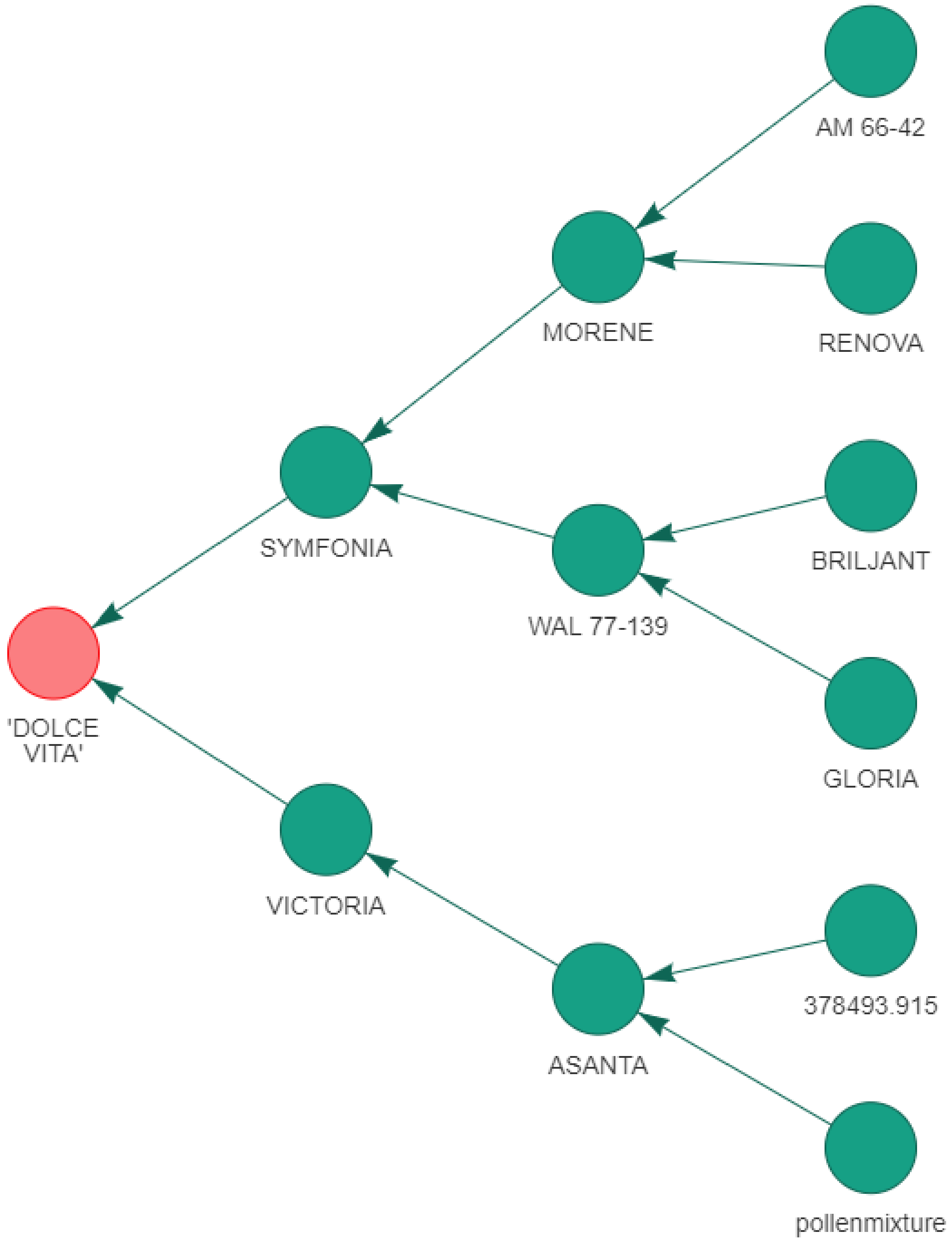

Supplementary Figure 3: Pedigree of ‘Dolce Vita’ obtained from the Potato Pedigree Database. Red nodes represent the parents used in the crosses for the this study.

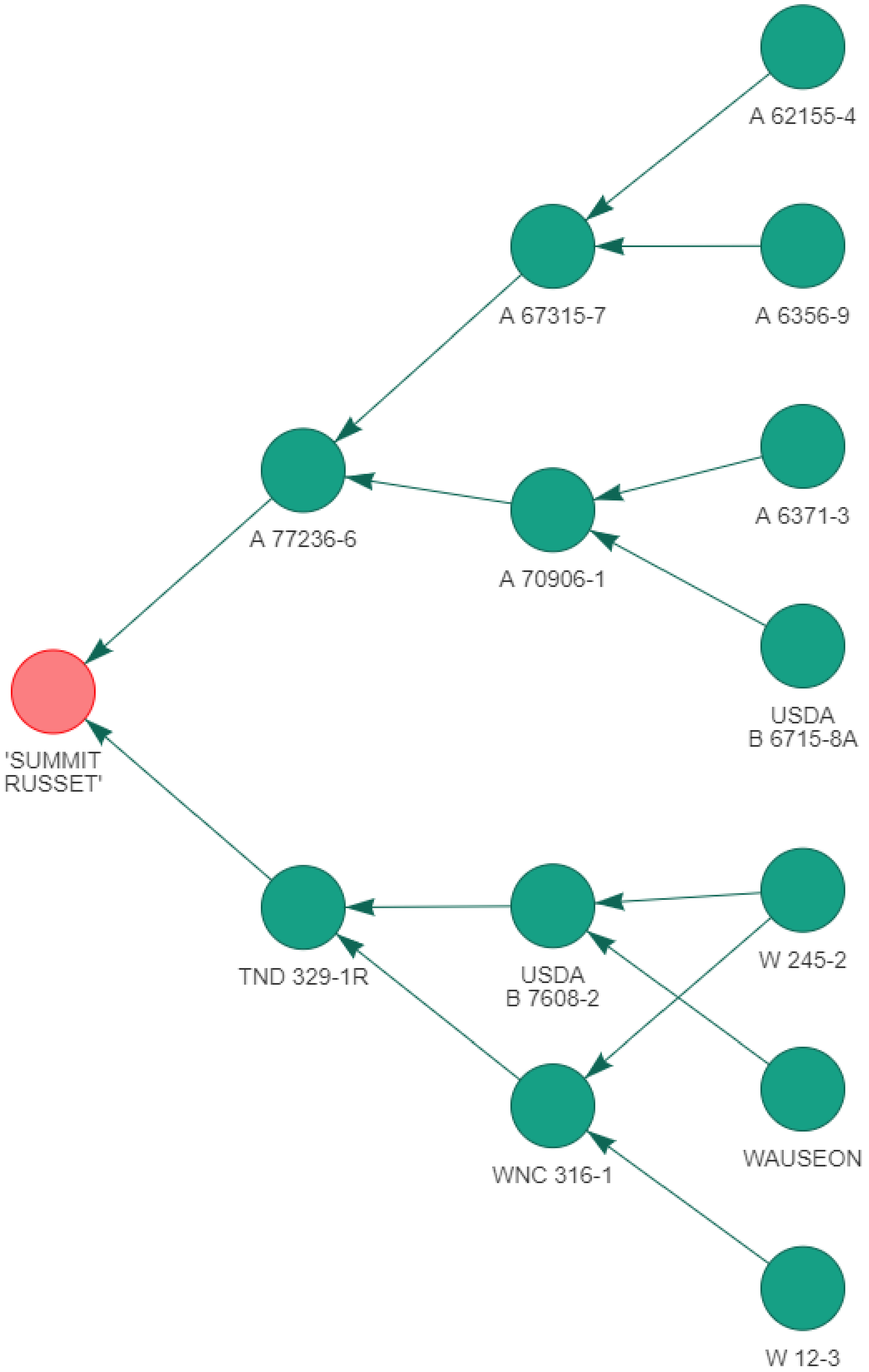

Supplementary Figure 4: Pedigree of ‘Summit Russet’ obtained from the Potato Pedigree Database. Red nodes represent the parents used in the crosses for the this study.

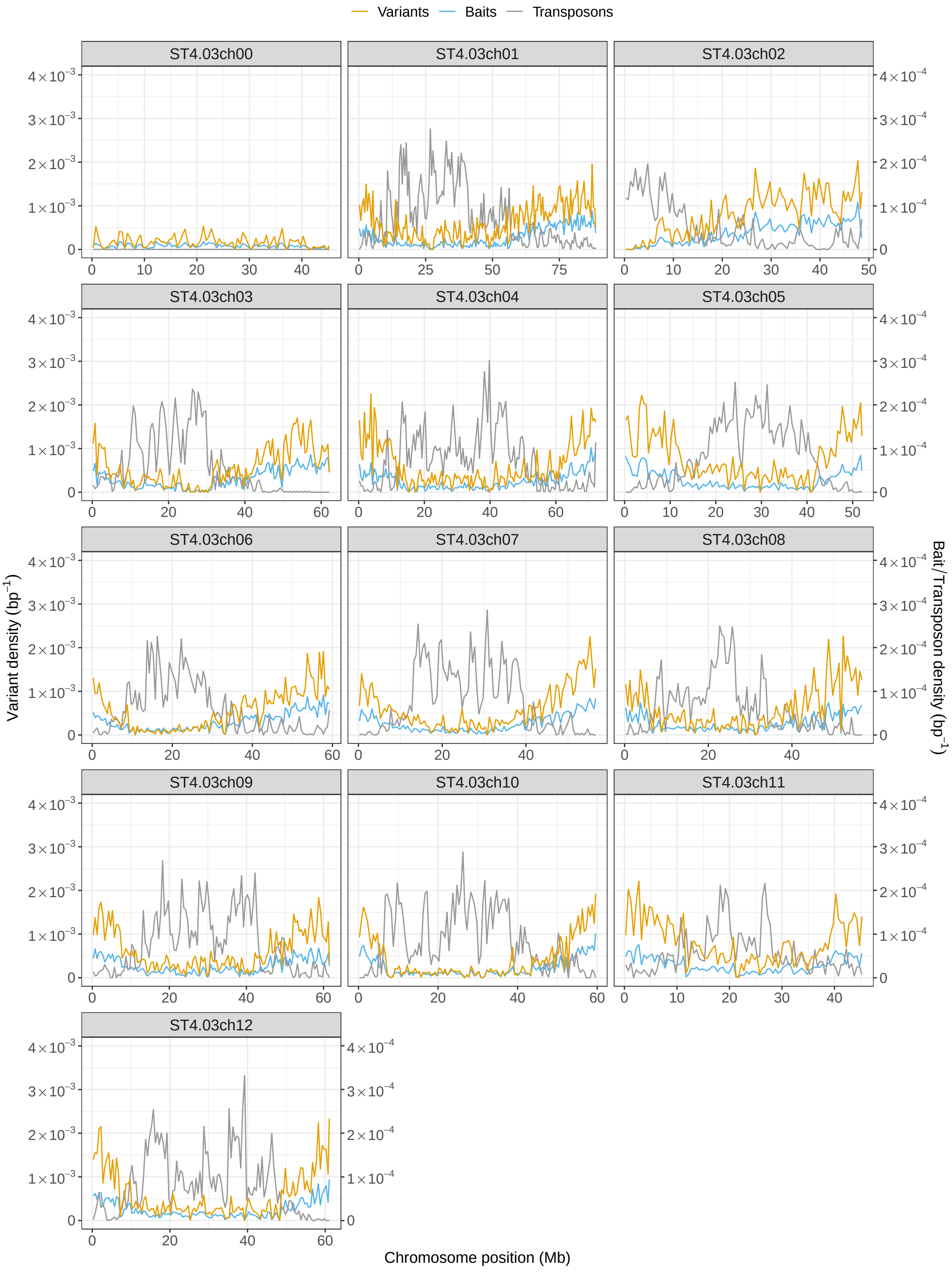

Supplementary Figure 5: Distribution of 454,247 variants (orange), 20,035 baits (blue) and 53,242 transposons (grey) across the chromosomes of the potato genome (chromosome 0: unanchored sequences). Densities are computed for adjacent genomic regions of 0.5Mb. Higher transposon density reflects the location of the centromeres.

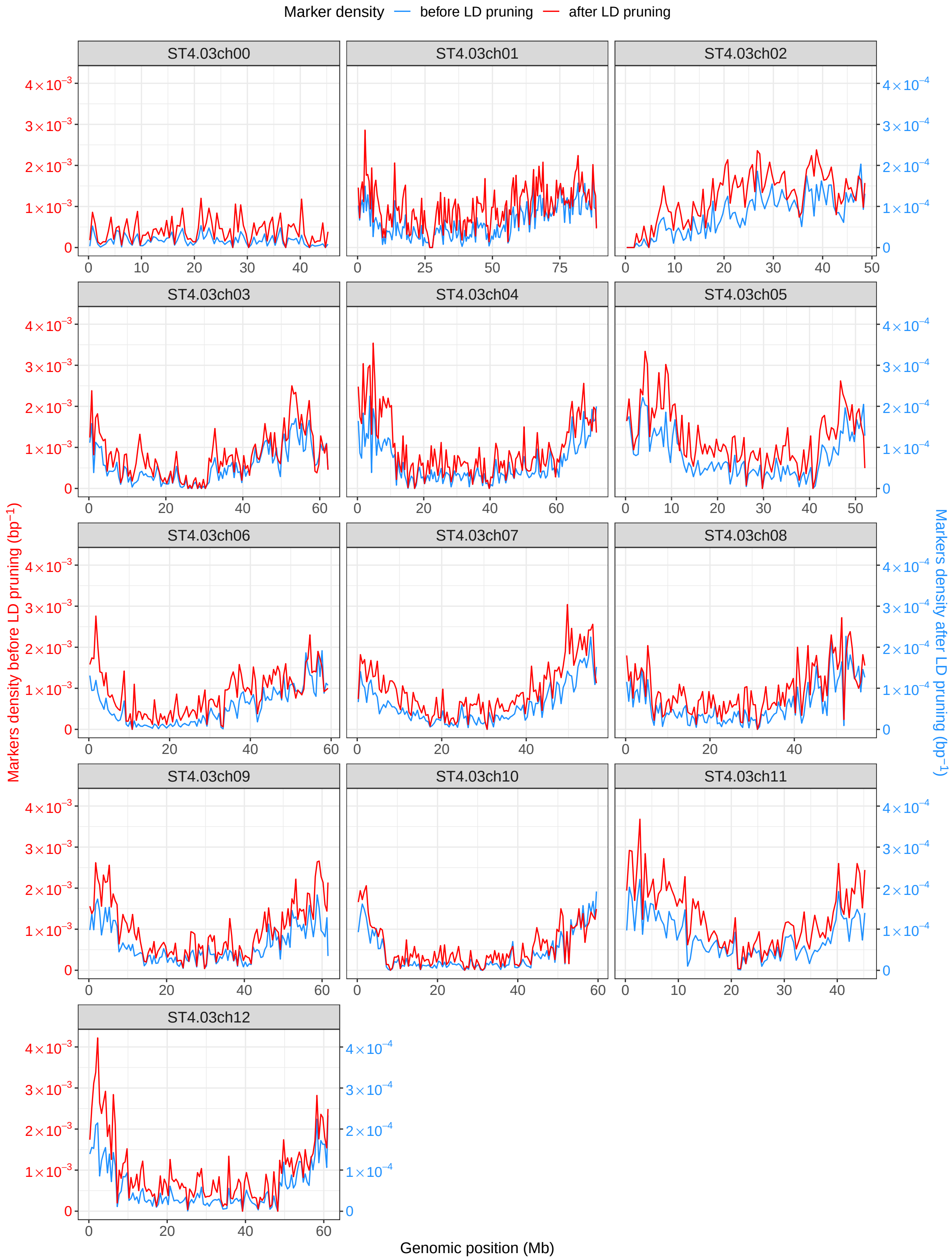

Supplementary Figure 6: Distribution of variants before (blue) and after (red) linkage disequilibrium pruning across the chromosomes of the potato genome (chromosome 0: unanchored sequences). Densities are computed for adjacent genomic regions of 0.5Mb.

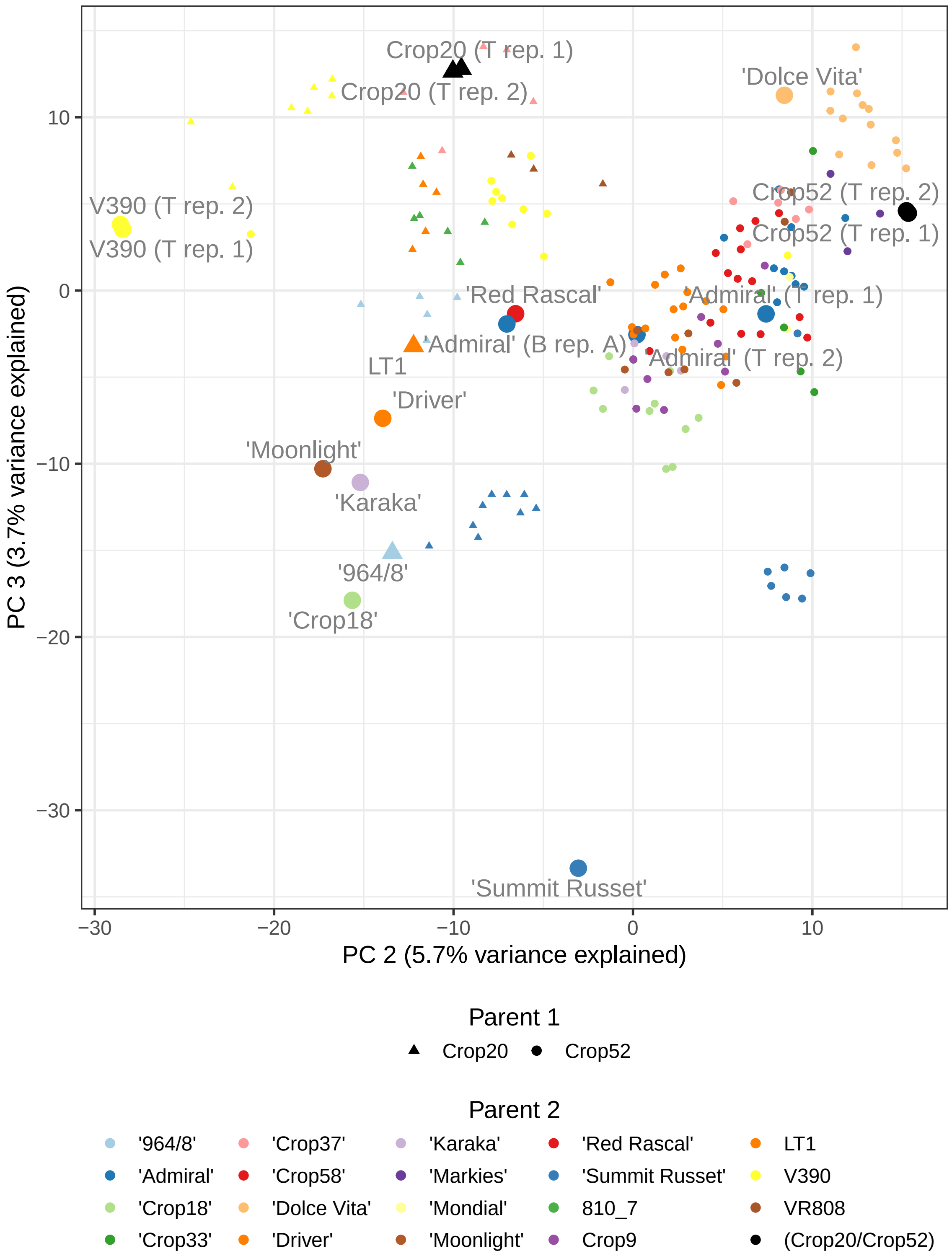

Supplementary Figure 7: PCA plot of the second and third principal components of a PCA applied to the variants most probable dosage for 176 samples. The name of the parent samples (large points) is indicated next to the corresponding point, while progeny samples are indicated with smaller points. For parent samples, 'T rep.' indicates a technical replicate, while 'B rep.' indicates a biological replicate. For the progeny samples, the shape of the points represent the first parent (i.e. Crop20 or Crop52), and the colour the second parent.

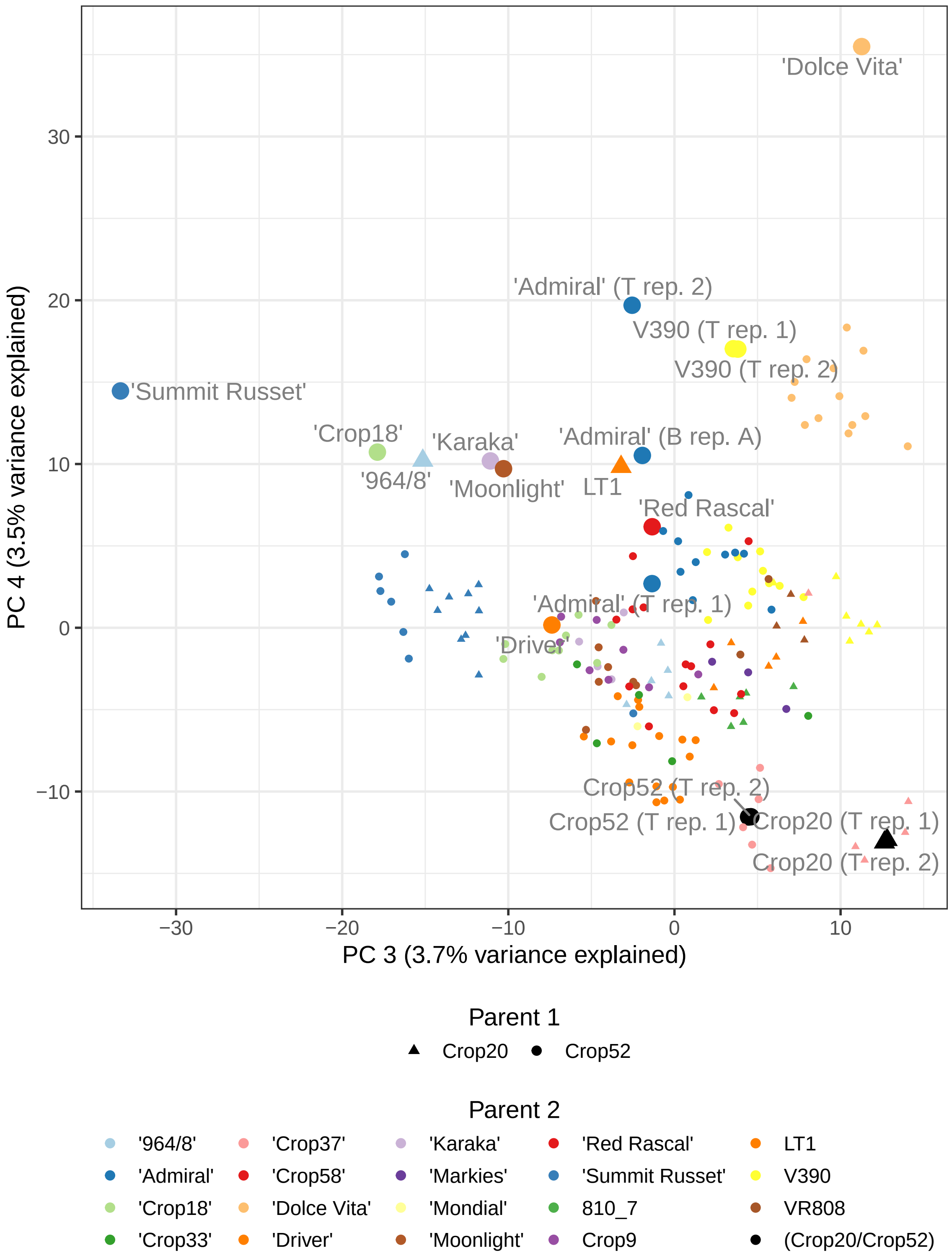

Supplementary Figure 8: PCA plot of the third and fourth principal components of a PCA applied to the variants most probable dosage for 176 samples. The name of the parent samples (large points) is indicated next to the corresponding point, while progeny samples are indicated with smaller points. For parent samples, 'T rep.' indicates a technical replicate, while 'B rep.' indicates a biological replicate. For the progeny samples, the shape of the points represent the first parent (i.e. Crop20 or Crop52), and the colour the second parent.

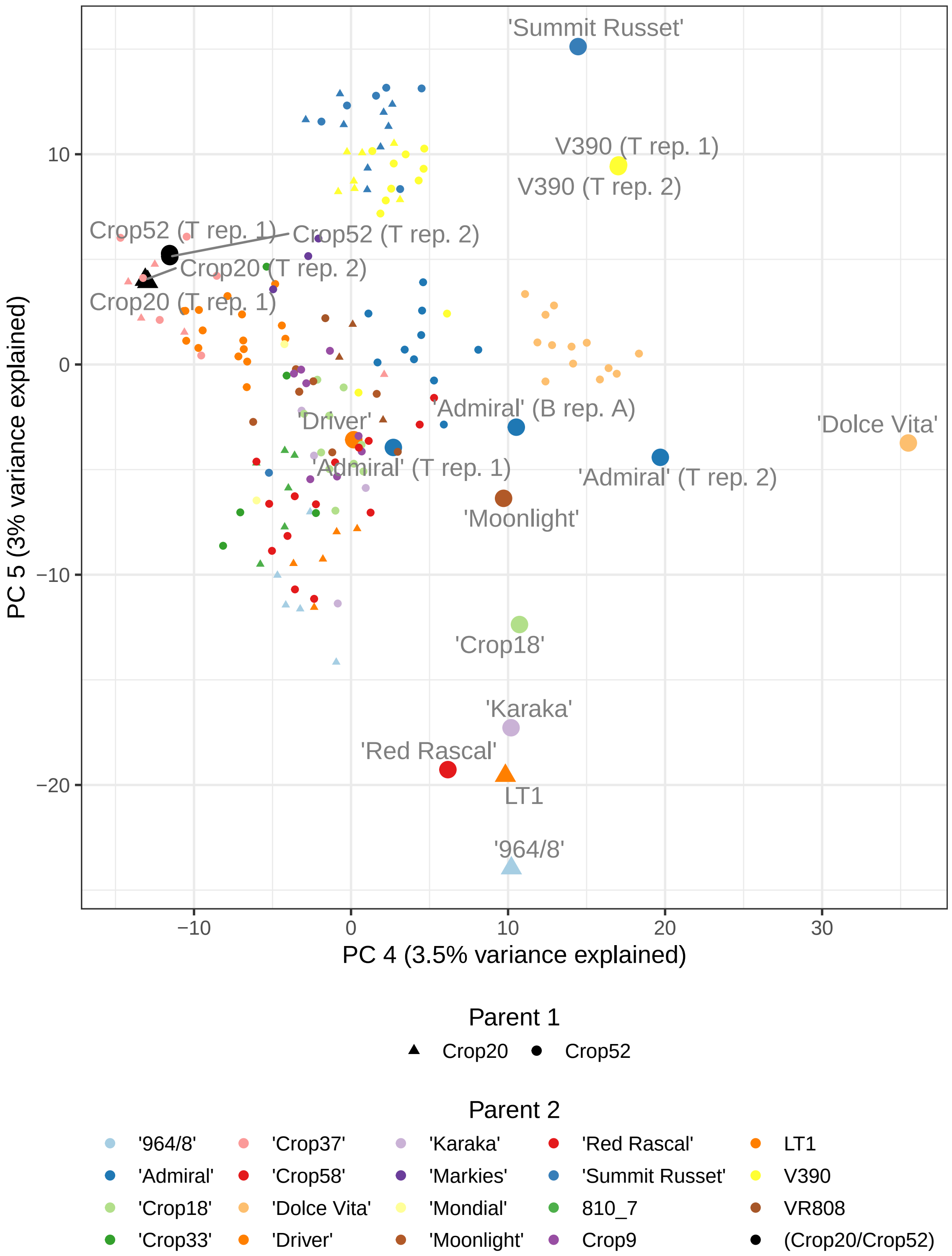

Supplementary Figure 9: PCA plot of the fourth and fifth principal components of a PCA applied to the variants most probable dosage for 176 samples. The name of the parent samples (large points) is indicated next to the corresponding point, while progeny samples are indicated with smaller points. For parent samples, ‘T rep.’ indicates a technical replicate, while ‘B rep.’ indicates a biological replicate. For the progeny samples, the shape of the points represent the first parent (i.e. Crop20 or Crop52), and the colour the second parent.

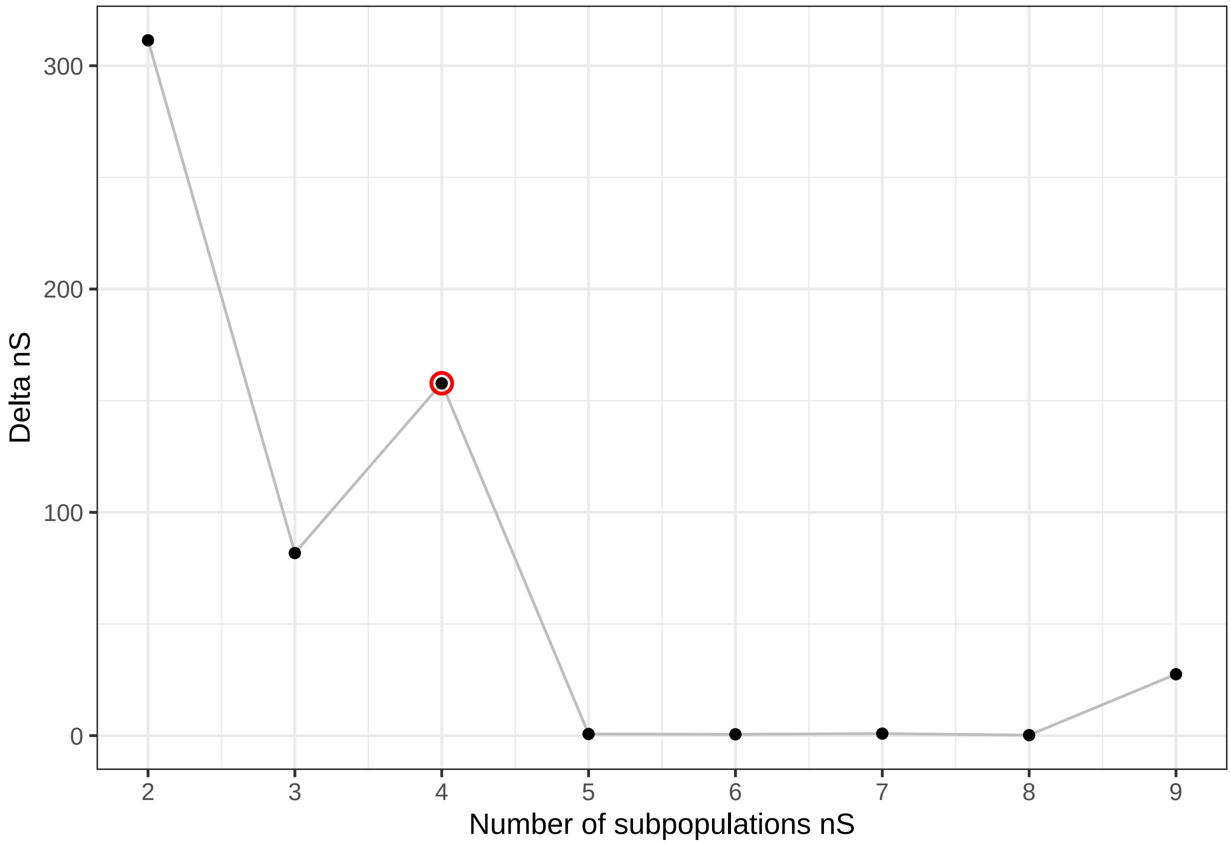

Supplementary Figure 10: ΔnS values for different values of the number of subpopulations nS used in STRUCTURE. For a given nS value, ΔnS is computed as the ratio of the average (computed over the different STRUCTURE runs with this value of nS) of the second derivative of the likelihood, over the likelihood variance (computed across the different STRUCTURE runs with this value of nS). The selected value of nS is circled in red.

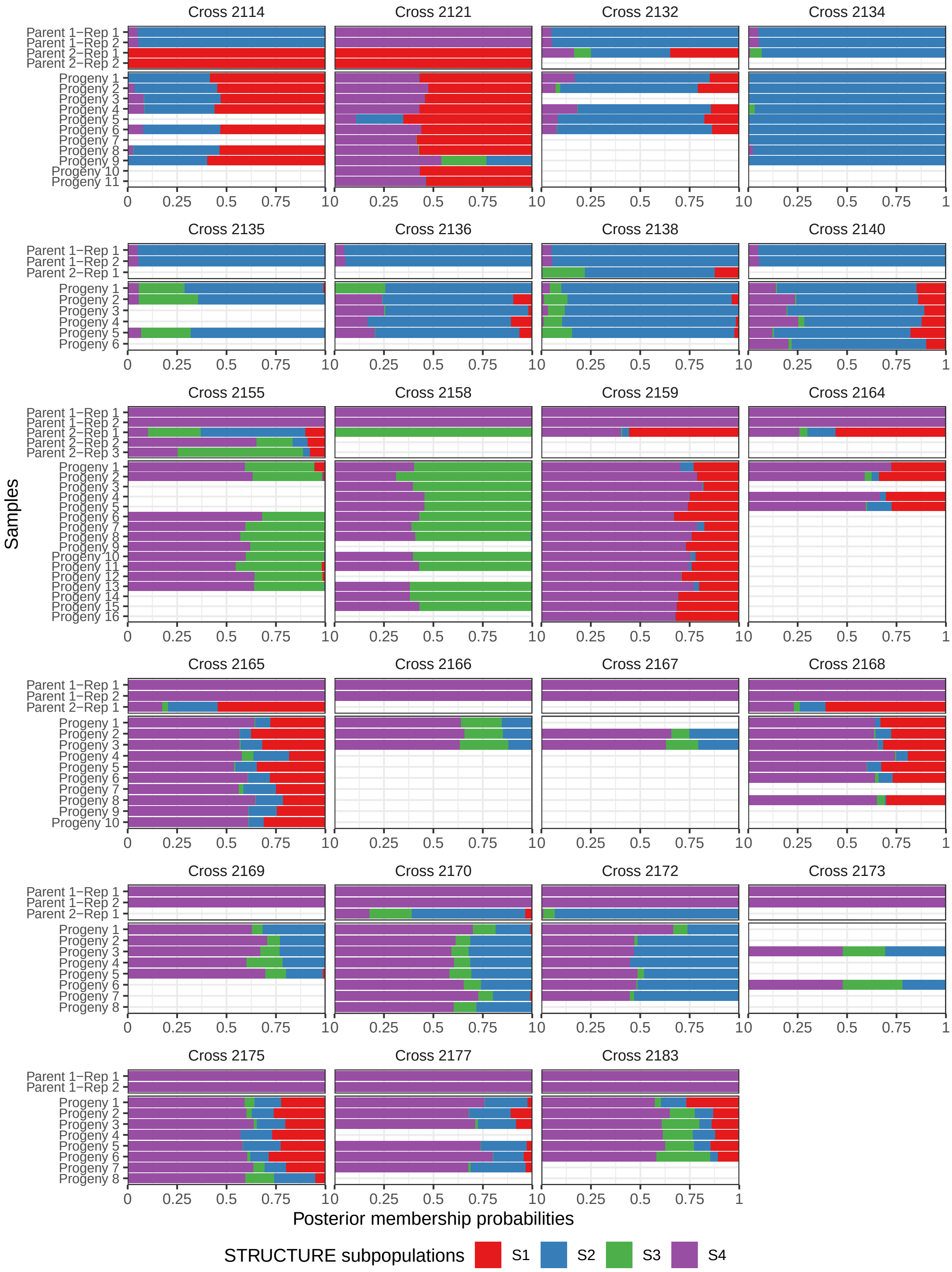

Supplementary Figure 11: STRUCTURE posterior membership probabilities of the parent and progeny samples from the different crosses. Missing progeny were culled in the breeding pipeline.

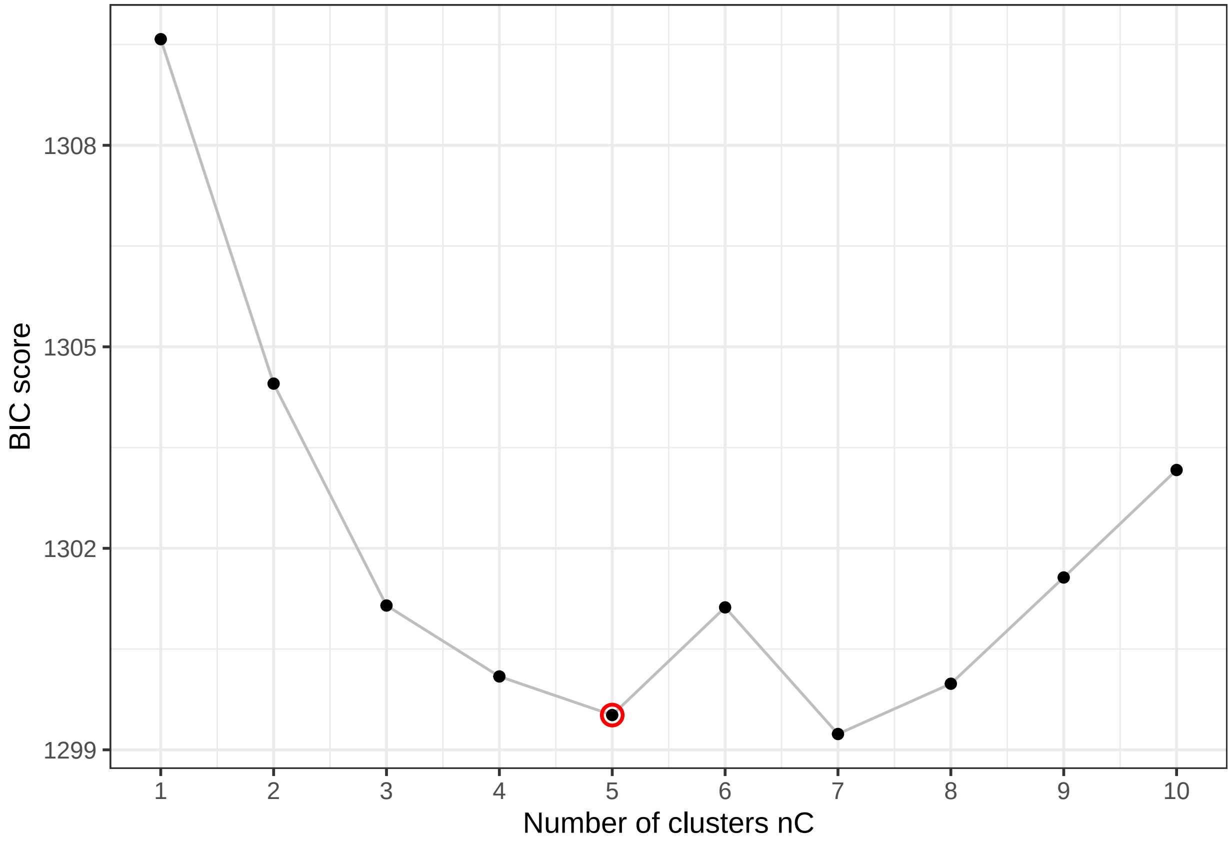

Supplementary Figure 12: BIC score obtained through k-means clustering for DAPC with different values for the number of clusters nC. The selected value of nC is circled in red.

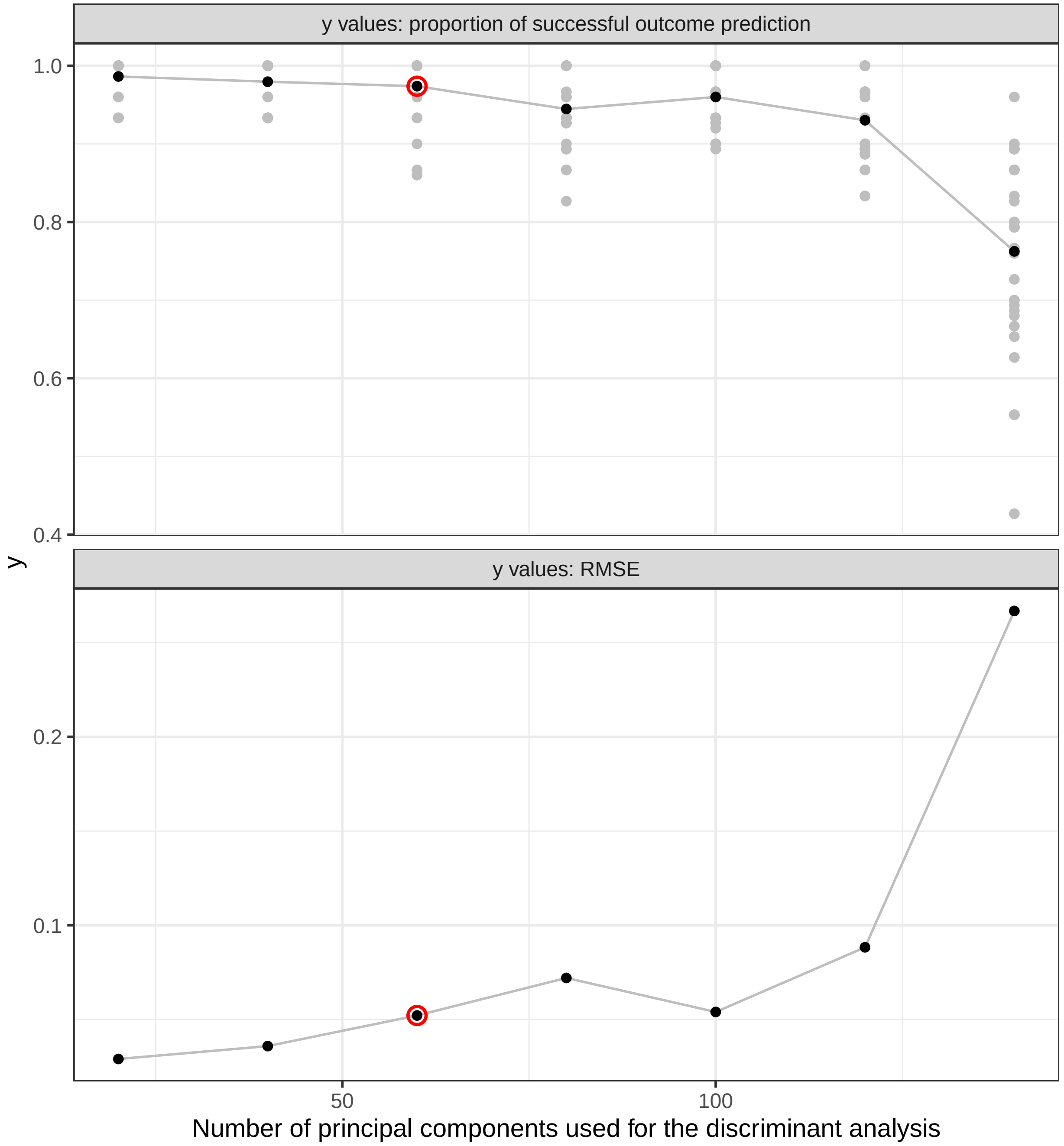

Supplementary Figure 13: Results of a cross-validation to assess the optimal number of principal components to use in the discriminant analysis for DAPC. For different numbers of principal components used, the top panel of the plot shows the average prediction accuracy in black, with accuracy of individual folds of the cross-validation shown in grey. The bottom panel shows the RMSE of the discriminant analysis for different number of principal components used. The selected value is circled in red.

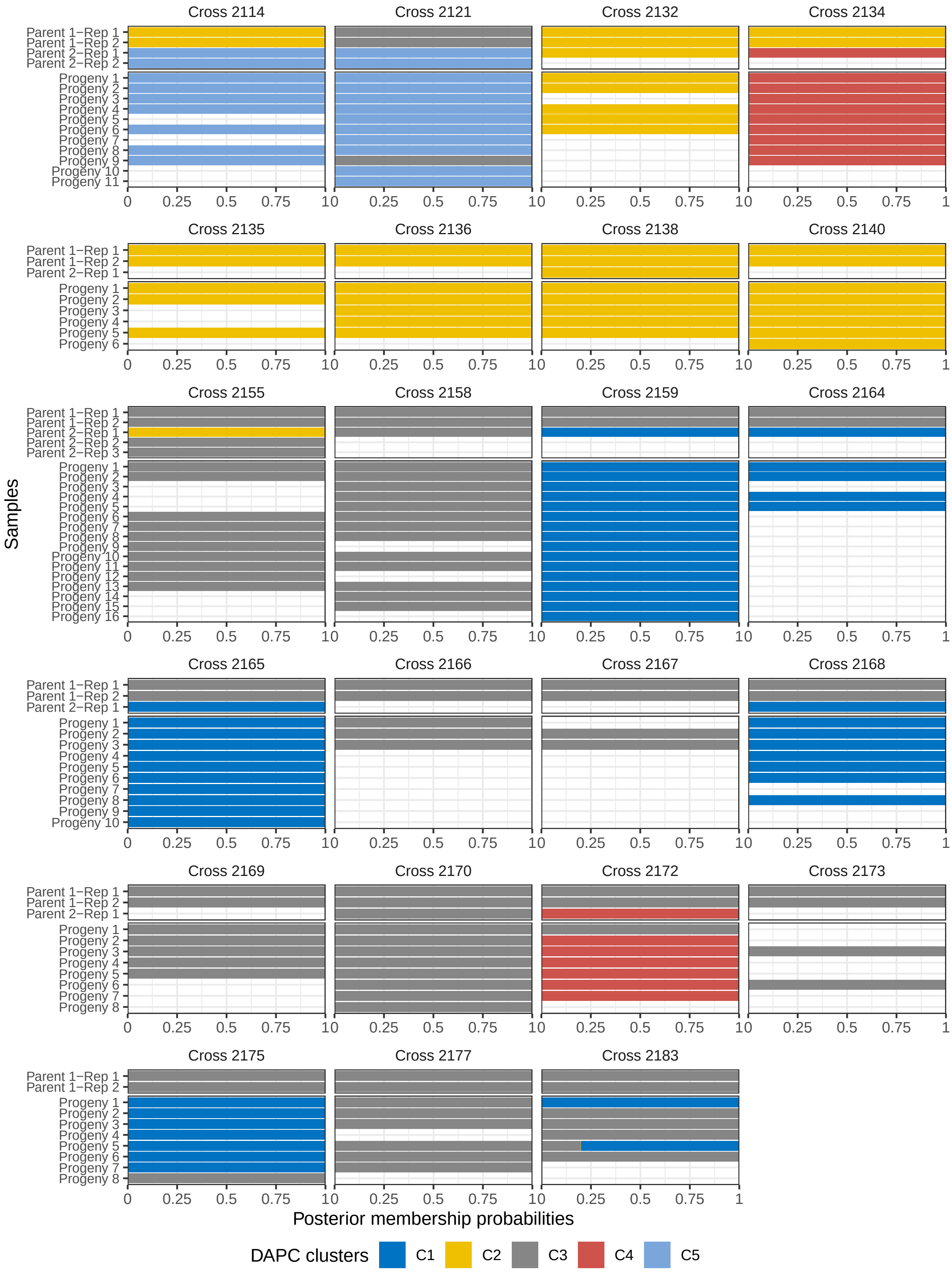

Supplementary Figure 14: DAPC posterior membership probabilities of the parent and progeny samples from the different crosses. Missing progeny were culled in the breeding pipeline.

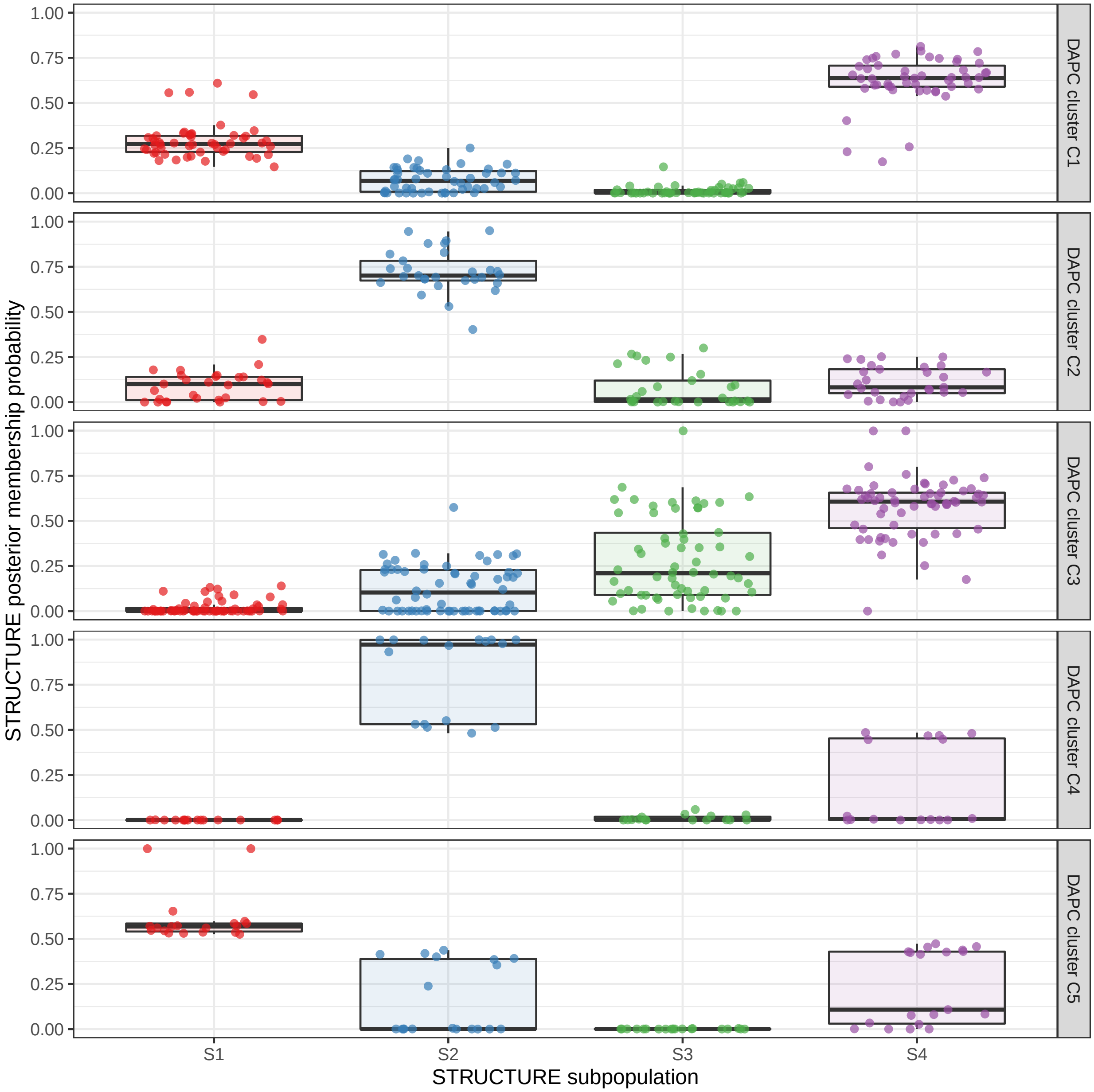

Supplementary Figure 15: Distribution of the STRUCTURE posterior membership probabilities of the samples for each DAPC cluster.

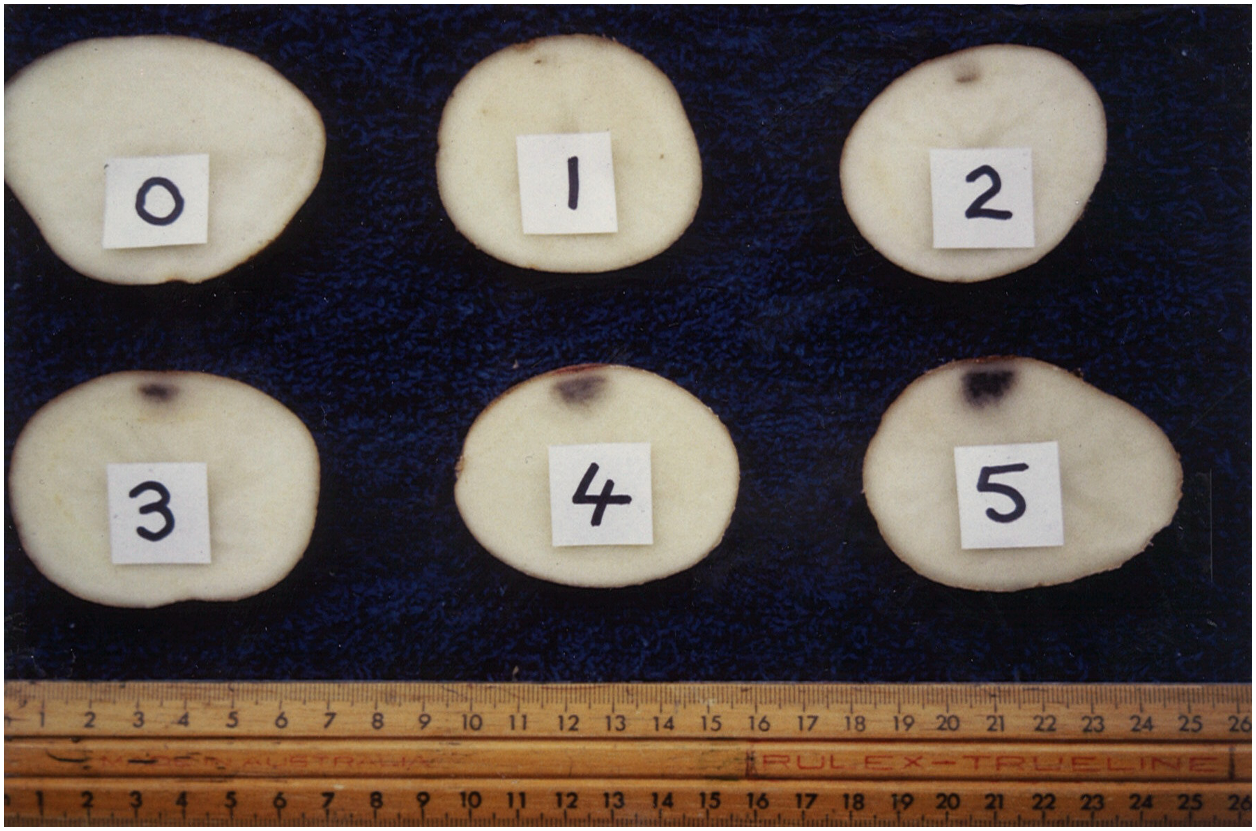

Supplementary Figure 16: Visual scale used for the scoring of tuber bruising.

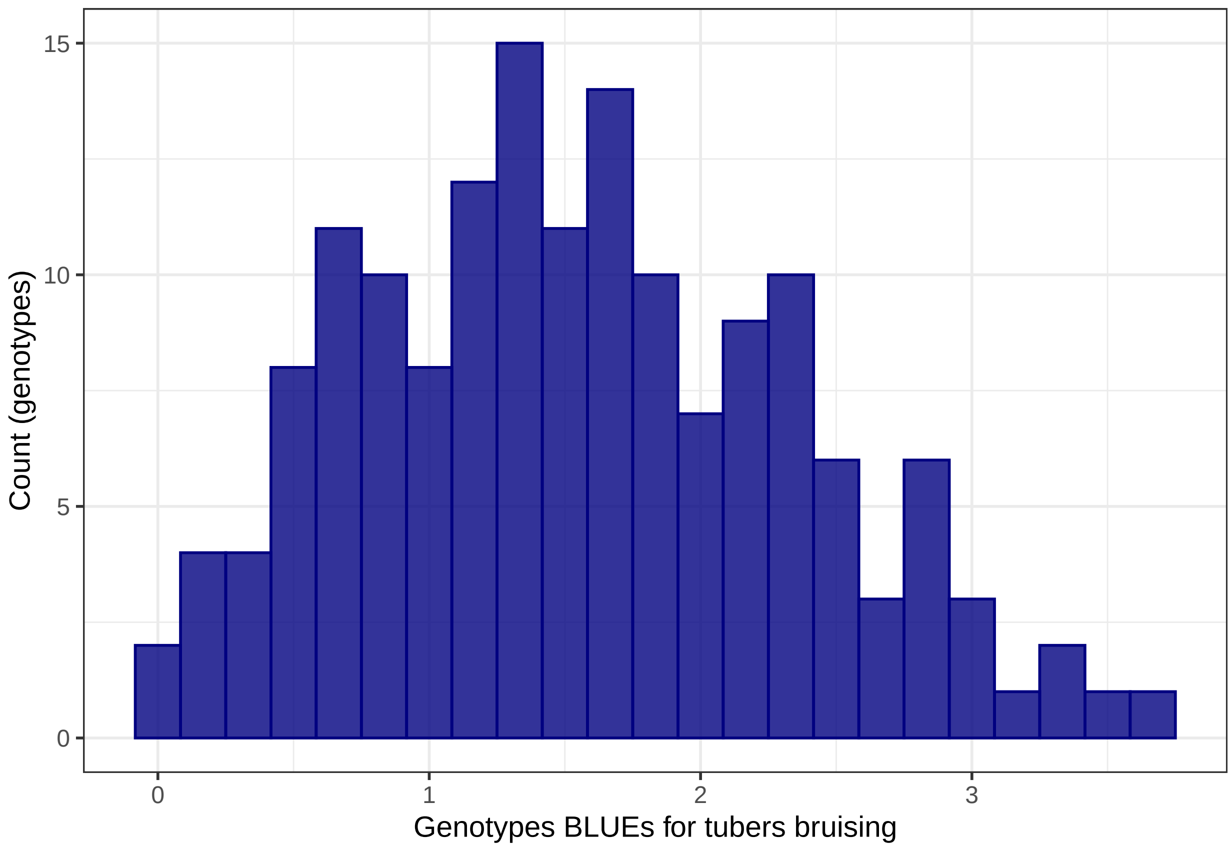

Supplementary Figure 17: Distribution of the bruising score Best Linear Unbiased Estimators (BLUEs) across the 158 progeny genotypes.

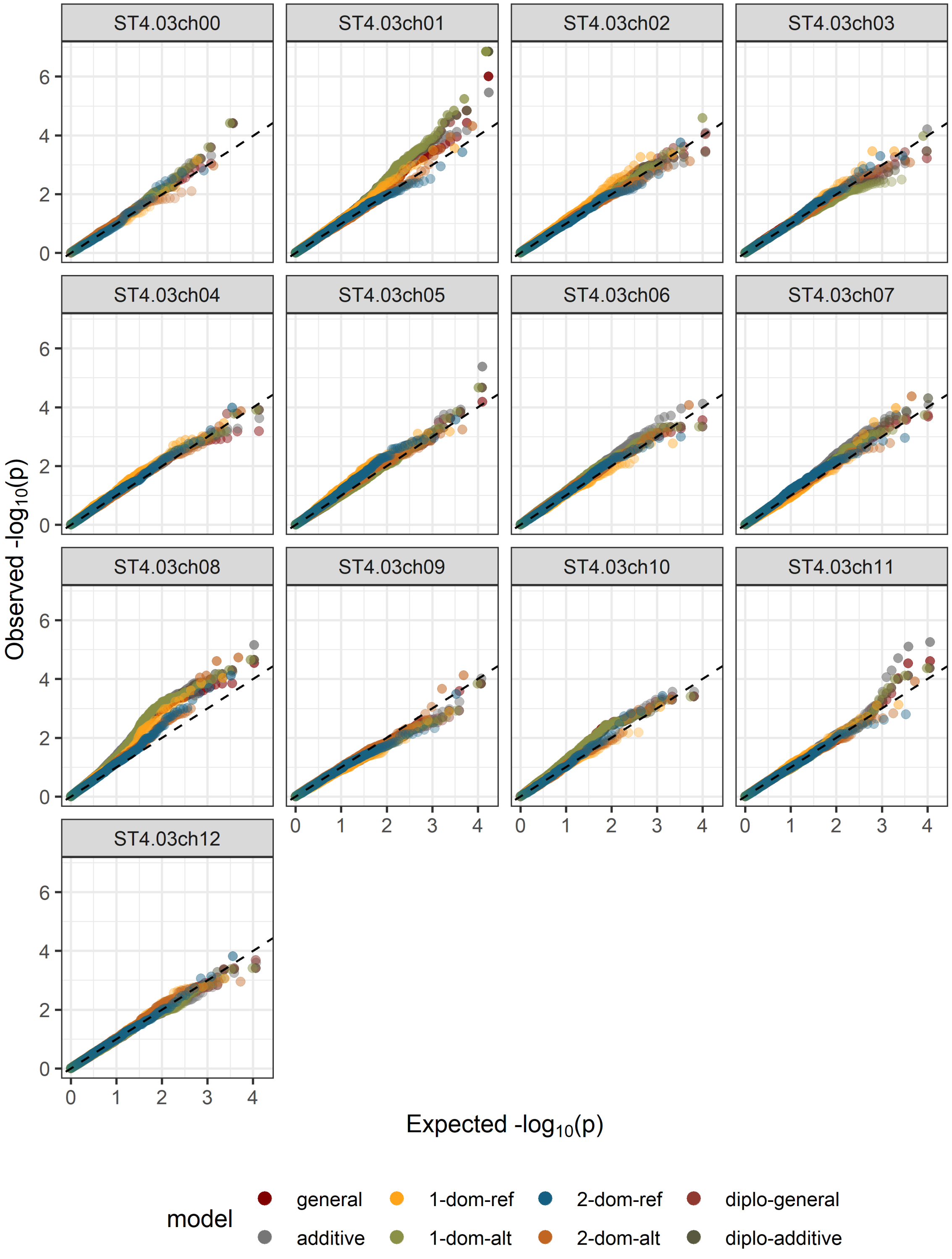

Supplementary Figure 18: QQ-plots of GWAS marker scores across the chromosomes of the potato genome for the K + Q structure population setting. Each colour represents a different genetic model.
